# Multiple source locations and long-distance dispersal explain the rapid spread of a recent amphibian invasion

**DOI:** 10.1038/s41437-025-00766-w

**Published:** 2025-05-16

**Authors:** Teun Everts, Io Deflem, Charlotte Van Driessche, Sabrina Neyrinck, Tom Ruttink, Hans Jacquemyn, Rein Brys

**Affiliations:** 1 Research Institute for Nature and Forest, Genetic Diversity, Geraardsbergen, Belgium; 2 KU Leuven, Department of Biology, Plant Conservation and Population Biology, Heverlee, Belgium; 3 Ghent University, Department of Biology, Terrestrial Ecology Unit, Ghent, Belgium; 4 Flanders Research Institute for Agriculture, Fisheries and Food, Plant Science Unit, Melle, Belgium; 5 Ghent University, Department of Plant Biotechnology and Bioinformatics, Ghent, Belgium

**Keywords:** Invasive species, Freshwater ecology

## Abstract

Rapid range expansions are characteristic for non-native invasive species when introduced outside their native range. Understanding the dynamics and mechanisms of expanding non-native invasive species is key for regional management. While population genetics and long-term occurrence records are often used in this context, each provides only partial insights, highlighting the need for a combined approach. We demonstrate this synergy using the American bullfrog (*Lithobates catesbeianus*) invasion in the Grote Nete river valley (Belgium) as a case study. It is commonly believed that this invasion constitutes a single metapopulation established by one primary introduction followed by downstream dispersal. However, recent evidence suggests a more complex scenario, involving introduction at multiple locations and bidirectional dispersal. To differentiate between both scenarios, we analysed nearly three decades of occurrence records and 8592 single nucleotide polymorphisms across 372 individuals from 31 localities, and determined the number of source locations, the range expansion rate, the population genetic structure, and the magnitude and direction of gene flow. We found that invasive spread originated from up to six source locations followed by bidirectional dispersal and downstream long-distance dispersal (LDD) events. Our results suggest that at least two source locations were founded by primary introductions, two from LDD events, while the remaining resulted from secondary introductions. A canal crossing the river was identified as a dispersal barrier, leading to different invasion dynamics on both sides. Our study shows how asynchronous introductions at multiple locations, dispersal barriers, and environmental heterogeneity can lead to distinct spread dynamics within a seemingly continuous and interconnected metapopulation.

## Introduction

Species distributions are naturally constrained by geographic boundaries and eco-evolutionary processes (Gaston 2009; Sexton et al. 2009). An increasing number of species are introduced outside their natural distribution areas, often driven by human activities (Channell and Lomolino 2000; Parmesan and Yohe 2003). The subsequent spread of these species in novel regions during biological invasions provides an opportunity to study the ecological and genetic processes driving rapid range expansions (Beer et al. 2024). As biological invasions are major drivers of global biodiversity loss (Clavero and García-Berthou 2005; Hogue and Breon 2022), it is important to develop a thorough understanding of the spatiotemporal invasion dynamics and mechanisms affecting the expansion of non-native species (Wilson et al. 2009).

Biological invasions are often portrayed as a linear, unidirectional process that generally includes a fixed sequence of three distinct stages: introduction, range expansion, and saturation (Andow et al. 1990). The spatial spread and cumulative abundance of non-native species during these stages are largely dictated by reproductive rate, density dependence, dispersal, and environmental conditions (Burton et al. 2010; Chuang and Peterson 2016), often following a sigmoid curve (Haubrock et al. 2022). During the introduction stage, small initial population sizes often limit rapid population growth and therefore spatial spread (Crooks 2005). Successful invaders subsequently enter the range expansion stage, which is characterised by increases in species cumulative abundance and spatial spread, as individuals move away from a region that has been established long enough for population dynamics to have reached an equilibrium (Chuang and Peterson 2016). As the ecosystem’s carrying capacity is approached, the cumulative abundance and population growth rate gradually decline, leading to the saturation stage (Andow et al. 1990). Empirically discriminating between these stages can inform on the expected range expansion speed and the factors governing spread (Burton et al. 2010; Fraser et al. 2015), but this is not straightforward. Each stage is influenced by abiotic and biotic factors (Shigesada and Kawasaki 2016) and can experience time lags, the duration of which are taxon- and environment-dependent (Crooks 2005; Spear et al. 2021). Additionally, not all invasions originate from a small number of founders (Signorile et al. 2014) nor do they always result from a single introduction event (Roman and Darling 2007). Multiple introductions may lead to the formation of spatially distinct satellite populations, which expand independently and may eventually merge, generating complex and unique patterns of spatial spread (Shigesada and Kawasaki 2016). Moreover, invasions do not necessarily expand their invaded range at a steady pace (Urban et al. 2008), nor do they always reach a saturation stage, as species may adapt to changing environmental conditions (Beer et al. 2024). Even within an invaded area, different invasion stages and spread rates can occur concurrently due to multiple asynchronous introductions, dispersal barriers, environmental heterogeneity, or unique eco-evolutionary dynamics (Berthouly-Salazar et al. 2013; Fraser et al. 2015; Haubrock et al. 2022). Consequently, the spread of non-native species is a complex and context-dependent process, which limits its generalisability and necessitates case-specific research.

Given the limited availability of long-term species monitoring data (Pergl et al. 2020), occurrence records sourced from citizen-science initiatives provide a valuable alternative for reconstructing invasion histories and assessing small-scale spread dynamics (Ceschin et al. 2018). Historical occurrence records can inform on the chronology of multiple introductions (Ryan et al. 2019) and the role of natural dispersal in promoting past and contemporary range expansions (Beer et al. 2024; Sherpa et al. 2020). Geographic profiling algorithms (geoprofiling hereafter) can be used to identify regions within the invaded range from which invasive spread most likely started, based on locations of occurrence records and a dispersal kernel (Le Comber and Stevenson 2012; Verity et al. 2014). Such regions, hereafter referred to as source locations, may have originated from primary introductions (i.e., initial introductions from the native range), secondary introductions (i.e., post-introduction human-mediated displacement), or natural long-distance dispersal (LDD) events. As such, geoprofiling analyses can help distinguish between single-source and multiple-source invasions—that is, whether non-native species began spreading from a single introduction point or multiple ones—as well as between short-distance natural dispersal and either LDD events or secondary introductions. While citizen-science data are straightforward, cost-effective, and cover large spatiotemporal scales, they may be insufficient to independently generate accurate and comprehensive insights, mainly due to biased data collection (Pocock et al. 2024).

Population genetic analyses offer unique insights into the history and spread dynamics of biological invasions, and can complement findings derived from historical occurrence records (Le Roux 2022; Sherpa and Després 2021). Population genetics are typically used to identify the provenance of non-native invasive species, estimate the minimum number of primary introductions, and delineate regions within the introduced range founded by individuals from genetically similar provenances (Matheson and McGaughran 2022; Sard et al. 2023). However, analysing the spread dynamics of biological invasions from a molecular perspective presents two main challenges. First, distinct source locations that result from multiple primary or secondary introductions of individuals with genetically similar origins cannot be distinguished (Mothes and Searcy 2024; Ryan et al. 2019). This can lead to an underestimation of the minimum number of primary introductions and may result in misattributing independent introduction events to natural dispersal processes (Welles and Dlugosch 2018). Second, genetic differentiation between introduced populations established by primary introductions from genetically distinct origins makes it difficult to separate founder effects from secondary introductions and natural dispersal (Sherpa and Després 2021). These issues can lead to superficial or even misguided understandings of invasion history and invasive spread rates. Inferential methods for spatiotemporal spread dynamics would therefore benefit from combining analyses of both population genetic and occurrence data (Estoup et al. 2010; Lu et al. 2023; Sherpa and Després 2021).

In this study, we combine geoprofiling based on citizen science occurrence records with detailed population genetic analyses to investigate the spread dynamics of the American bullfrog (*Lithobates catesbeianus*) in the Grote Nete river valley (Belgium). Since their introduction in the 1990s, bullfrogs have rapidly spread, yet the factors driving this expansion remain poorly understood. It is commonly believed that this invasion constitutes one continuous, interconnected metapopulation established by a single primary introduction followed by downstream dispersal (Descamps and De Vocht 2023). However, recent evidence suggests a more complex invasion history, involving multiple introductions, either primary or secondary, and bidirectional, discontinuous dispersal (Everts et al. 2023). To distinguish between both scenarios, we reconstructed the historical course of bullfrogs invading the Grote Nete river valley, identified potential source locations, and assessed the magnitude and direction of gene flow.

## Materials and methods

### Study species

**Fig. 1:**
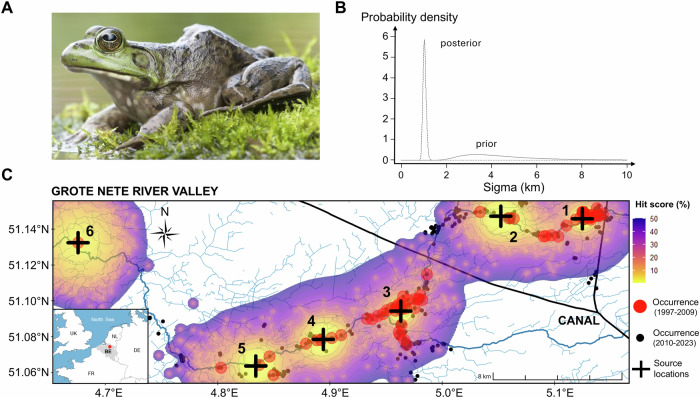
Geoprofiling of the American bullfrog invasion in the Grote Nete river valley using citizen-science occurrence records. **A** Adult female American bullfrog, courtesy of Vildaphoto/Rollin Verlinde. **B** Prior and posterior distributions describing bullfrog natural dispersal analysing occurrence records between 1997 and 2009. The mean posterior value of the standard deviation in dispersal distance (sigma) was estimated at 1.05 ± 0.070 km. **C** The top 50% hit scores (i.e., proportion of the area that must be searched before the true source location is found) determined by the geoprofiling algorithm. Areas characterised by low hit scores indicate locations most likely to contain a source location, and are highlighted in yellow. The model supports six source locations, indicated as numbered black crosses. Occurrence records used for geoprofiling are shown as red circles, while all other occurrence records are shown as black circles (Fig. S1). Canals under which the Grote Nete river passes via a culvert are marked in black. The flow direction of the Grote Nete river is from east to west.

The American bullfrog (bullfrog hereafter) is a large pond-breeding amphibian native to eastern North America (Fig. 1A). Commercial frog farming and pet trade have led to their introduction to western North America, South America, Asia, and Europe throughout the nineteenth and twentieth centuries (Ficetola et al. 2007; Lever 2003). Established non-native bullfrog populations can negatively impact native amphibian species with a similar breeding phenology through predation, competition, and disease transmission (Everts et al. 2024; Hossack et al. 2023). Belgium is one of the European countries containing free-ranging bullfrog populations, mostly in the Northern region of the country (Flanders; Everts et al. 2023; Ficetola et al. 2007). Although the historical trajectory of the invasion in Flanders is undocumented, anecdotal evidence suggests two distinct modes of introduction. Primary introductions likely occurred when bullfrogs were unintentionally brought into the region as eggs or tadpoles hitchhiking with fish imports directly from their native range. Secondary introductions, on the other hand, refer to the subsequent human-mediated spread of bullfrogs within the region, when tadpoles were sold in pet markets and fish stores, leading to their release into outdoor garden ponds (Jooris 2005). While legal measures have prohibited the trade and possession of bullfrogs since 2009, established populations now have occupied an area of approximately 365 km^2^ (Everts et al. 2023). Aside from several satellite populations, the main concern from a management perspective is the Grote Nete river valley, which is formed by the Grote Nete river that crosses natural areas intertwined with residential areas. Here, semi-natural and artificial garden and fish ponds are abundantly present, which, due to their permanent nature, are habitats in which bullfrogs thrive (Everts et al. 2023).

### Citizen-science occurrence records

#### Data acquisition

Bullfrogs are well-suited for passive observation by the general public, as juveniles and male adults produce distinct sounds that are easily detectable. However visual identification can be challenging due to possible confusion with morphologically similar species, such as *Pelophylax* species. Occurrence records of invasive bullfrogs in Belgium were downloaded from GBIF (https://gbif.org) on November 3rd, 2023. Following the guidelines outlined by Pocock et al. (2024) for addressing potential misidentifications, we excluded occurrence records that were not expert-verified. This yielded 1585 occurrences recorded between 1997 and 2023 (Fig. S1).

#### Geographic profiling

To estimate the total number and approximate regions of source locations (i.e., regions within the invaded range from which invasive spread most likely began), we conducted a geoprofiling analysis using occurrence records (Le Comber and Stevenson 2012). Given the likelihood of the species being introduced at multiple locations, as hypothesised in our alternative invasion scenario (Everts et al. 2023), we applied the Dirichlet-Process-Mixture (DPM) method. This Bayesian algorithm is capable of detecting multiple source locations when present (Verity et al. 2014) and demonstrates comparable accuracy when applied to opportunistic occurrence records as with systematic survey data (Faulkner et al. 2017). To minimise bias from clustered records, which can distort model outputs (Verity et al. 2014), measures were implemented to reduce the initial set of retrieved occurrences. Only occurrences recorded up until 2009 were used (*n* = 430 records remaining), based on the assumption that deliberate releases significantly declined following the legal prohibition of bullfrog possession and trade introduced that year. Thus, observations after 2009 were presumed to result primarily from natural dispersal rather than additional human-mediated introductions (Fig. S1). When multiple occurrences were recorded at identical water bodies or within a range of 200 metres, we only retained the earliest record as geoprofiling does not account for the chronological order of observations. The final dataset used for geoprofiling comprised a total of 92 occurrence records.

We implemented the DPM model in RStudio version 4.3.0 (RStudio Team 2023), using a modification of the Rgeoprofile R package (https://github.com/bobverity/Rgeoprofile). The DPM model is informed by a prior distribution of sigma, representing a dispersal kernel of the target species (i.e., the standard deviation of the bivariate normal distribution describing dispersal from a source). We set the mean of the prior distribution of sigma to 5 km, meaning that over this 12 year period (1997–2009), the majority of natural bullfrog dispersal (68%, 95%, and 99%) was expected to occur within 5, 10, and 15 km of their source location, respectively, in line with the properties of a normal distribution. To account for potential natural long-distance dispersal events, such as passive dispersal of eggs and tadpoles during floods, we set the variance of the prior distribution of sigma to 10 km^2^, and the shape parameter to 1.5, promoting an inverse-gamma, right-skewed distribution (Faulkner et al. 2017; Fig. 1B). The resulting prior ranges from a sigma value of 1.5 km (corresponding to 0.125 km/year) to 10 km (0.833 km/year), with the highest frequency around 2.5 km (0.208 km/year). Although bullfrogs can actively move up to 1.6 km each year (Sepulveda and Layhee 2015; Smith and Green 2005), consistent, unidirectional movement is unlikely (Descamps and De Vocht 2016), particularly given the availability of many suitable, yet uncolonized habitats during the early stages of invasion (Arim et al. 2005). The actual systematic annual displacement of bullfrogs is therefore expected to be substantially lower than 1.6 km in a year. The predefined dispersal kernel represented by this prior distribution therefore aligns well with the described dispersal behaviour of bullfrogs, both within our study system (Descamps and De Vocht 2016) and in other invaded ranges (Sepulveda and Layhee 2015; Smith and Green 2005). Regardless, the model is designed so that the effect of the prior is minimal, especially in data-rich contexts, which is essential given uncertainties surrounding dispersal behaviour of non-native invasive species (Faulkner et al. 2017). Since the accuracy of the prior distribution cannot be directly assessed, the resulting mean sigma value and geographic profile should be interpreted with caution. We conducted the analysis with 1000 burn-in iterations, 10,000 sampling iterations, and five MCMC chains (Gelman-Rubin statistic was 1.00134).

#### Spatiotemporal invasion dynamics

We reconstructed past spatiotemporal invasion dynamics by analysing temporal increments in occurrence records (*n* = 1585). For every year since the first observation, we quantified the Extent of Occupancy (EOO) in square kilometres, which is a scaled metric that represents the area of potential suitable habitat currently occupied by a taxon (IUCN Standards and Petitions Committee 2022). Even though the EOO may be sensitive to spatial and temporal variations in sampling intensity, it can accurately represent a species’ occupied area for conspicuous taxa in well-sampled regions (IUCN Standards and Petitions Committee 2022). This is likely the case for the bullfrog invasion in Flanders, as (1) bullfrogs produce distinct calls, (2) they have invaded a densely populated area, and (3) large-scale monitoring started shortly after the first observation (Jooris 2005). Therefore, the EOO is unlikely to be significantly affected by spatial or temporal biases. We constructed circular perimeters with a 1 km radius around each observation of each year. This buffer conservatively represents the area in which that particular observation may be located within a year, based on the species’ dispersal capacity (Smith and Green 2005). For each year, overlapping buffers were merged, and the EOO was quantified by calculating the total area of the merged buffers. We further assumed that once bullfrogs have colonised a pond, it will remain occupied, and thus that EOO’s of successive years are cumulative (Everts et al. 2024). Then, we calculated the yearly range expansion speed (*E*) based on the year-by-year increase of the previously calculated EOO’s (*C*). The range expansion speed (in metres per year) in two successive years can be obtained using the following formula (Lensink 1997):

$$E=\sqrt{{C}\,{x}\,\frac{1}{\pi }}$$

### Population genetics

#### Field sampling

Bullfrog tissue samples were collected in the summers of 2021 and 2022. Individuals were captured using fyke nets in 31 ponds (hereafter referred to as localities; Fig. S2), covering the complete invaded range within the Grote Nete river valley (Everts et al. 2023). Both single and double fyke nets were used (for details see Everts et al. 2022). For each locality, a maximum of 20 individuals were selected for tissue sampling. When more individuals were captured, efforts were made to obtain a representative genetic sample of the locality while minimising bias from closely related individuals (Goldberg and Waits 2010; Fig. S3). In total, genetic material from 372 individuals was collected (Table 1). A small tail clipping of approximately 25 mm^2^ was collected from tadpoles using a sterilised scalpel and tweezers. For adult and juvenile individuals, 3 mm from the toe was collected. Extracted tissue samples were submerged in 1 mL of pure (96%) ethanol and stored in dark conditions at 4 °C.

**Table 1:** Details of the sampled bullfrog localities, including locality number, latitude and longitude, total number of sampled individuals (*N*), number of individuals retained after filtering for full-sibs (*N*_exclFS_), average expected (*H*_*e*_) and observed (*H*_*o*_) heterozygosity, inbreeding coefficient (*F*_*IS*_), mean relative immigration rate (*I*), mean relative emigration rate (*E*), and the immigration to emigration ratio (*R*_*I/E*_).

Locality	Latitude	Longitude	*N*	*N*_exclFS_	*H*_*e*_	*H*_*o*_	*F*_*IS*_	*I*	*E*	*R*_*I/E*_
1	51°08'38.8“N	5°09'22.3“E	14	9	0.2023	0.2116	−0.050	0.110	1.391	0.110
2	51°08'56.0“N	5°08'42.0“E	12	11	0.2439	0.2320	0.032	0.311	0.778	0.311
3	51°08'44.2“N	5°08'25.8“E	17	17	0.2413	0.2222	0.071	0.321	0.763	0.321
4	51°08'16.4“N	5°08'27.2“E	14	11	0.2130	0.2276	−0.053	0.157	0.911	0.157
5	51°11'12.1“N	5°09'30.2“E	14	12	0.2107	0.2121	−0.011	0.187	0.888	0.187
6	51°06'51.8“N	5°08'10.7“E	1	1	–	0.2553	–	–	–	–
7	51°07'24.2“N	5°07'06.6“E	14	10	0.1849	0.1747	0.045	0.136	0.133	1.023
8	51°07'59.5“N	5°06'36.0“E	16	14	0.2119	0.2010	0.043	0.182	0.185	0.984
9	51°08'13.6“N	5°06'11.9“E	16	9	0.2215	0.2011	0.073	0.201	0.206	0.976
10	51°08'11.4“N	5°05'45.6“E	2	2	0.2370	0.2233	−0.065	0.107	0.120	0.892
11	51°08'11.0“N	5°05'05.6“E	1	1	–	0.1921	–	–	–	–
12	51°08'49.2“N	5°03'52.6“E	5	4	0.1992	0.2466	−0.226	0.122	0.131	0.931
13	51°09'14.4“N	5°04'12.7“E	15	13	0.2255	0.2124	0.045	0.208	0.203	1.025
14	51°09'01.1“N	5°02'21.8“E	3	3	0.1910	0.2405	−0.267	0.095	0.103	0.922
15	51°08'55.7“N	5°01'46.6“E	17	15	0.2397	0.2285	0.037	0.255	0.241	1.058
16	51°09'00.4“N	5°00'57.2“E	15	11	0.2395	0.2407	−0.008	0.208	0.215	0.967
17	51°08'38.8“N	5°00'56.9“E	13	13	0.2361	0.2264	0.031	0.195	0.199	0.980
18	51°08'28.0“N	5°00'02.2“E	17	7	0.2103	0.2305	−0.085	0.108	0.152	0.711
19	51°05'58.6“N	4°58'54.1“E	19	14	0.2319	0.2364	−0.015	0.193	0.221	0.873
20	51°05'43.4“N	4°58'29.3“E	13	10	0.2340	0.2587	−0.091	0.177	0.217	0.816
21	51°05'44.5“N	4°57'23.8“E	18	14	0.2192	0.2157	0.016	0.183	0.206	0.888
22	51°04'34.3“N	4°59'18.6“E	1	1	–	0.1860	–	–	–	–
23	51°04'28.2“N	4°58'42.2“E	19	8	0.1860	0.1812	0.015	0.121	0.134	0.903
24	51°04'34.7“N	4°58'01.2“E	16	14	0.2139	0.2235	−0.032	0.184	0.203	0.906
25	51°05'27.2“N	4°55'58.4“E	12	10	0.2355	0.2015	0.119	0.129	0.132	0.977
26	51°04'50.2“N	4°53'03.1“E	15	8	0.2191	0.2200	−0.017	0.113	0.146	0.774
27	51°04'12.7“N	4°51'21.6“E	19	12	0.2182	0.1983	0.072	0.174	0.226	0.770
28	51°03'27.0“N	4°49'21.4“E	11	9	0.1815	0.1743	0.029	0.081	0.115	0.704
29	51°03'45.0“N	4°48'05.8“E	5	3	0.1803	0.2347	−0.300	0.075	0.133	0.564
30	51°04'00.1“N	4°46'10.6“E	15	10	0.2008	0.1903	0.038	0.142	0.157	0.904
31	51°08'16.1“N	4°36'25.2“E	3	2	0.1991	0.1836	−0.047	0.0568	0.116	0.490

Summary statistics for localities 6, 11, and 22 were omitted as only one individual per locality was sampled. Statistics per locality were calculated after excluding full-sibs. No statistics could be calculated for localities 6, 11, and 22 due to limited sampling size.

#### DNA extraction and library preparation

Genomic DNA was extracted from all collected tissue using the DNeasy Blood & Tissue kit (Qiagen). The concentration of DNA in each sample was quantified using a Promega Quantus™ fluorimeter, and normalised to a concentration of approximately 10 ng/µL. DNA quality was assessed on 1% agarose gels. The 372 specimens sampled from 31 localities (Table 1 and Fig. S2) were then genotyped at genome-wide single nucleotide polymorphisms (SNPs) sourced by Genotyping-by-Sequencing (GBS) using the double-digest GBS protocol (Elshire et al. 2011). The protocol for selecting restriction enzymes, library preparation, and bioinformatic processing are in Supporting Information 1 and Table S1. Since a large proportion of sampled individuals were tadpoles, we risked including a higher proportion of close relatives than expected under random sampling, which can bias parameter estimates (Goldberg and Waits 2010). One random individual per full-sib pair was therefore discarded (see ‘Estimating genetic connectivity’), resulting in a total of 285 retained individuals (Table 1 and Fig. S4).

#### Estimating genetic diversity and structure

For each sampled locality, observed heterozygosity (*H*_*o*_), expected heterozygosity (*H*_*e*_), and the inbreeding coefficient (*F*_*IS*_) were estimated using the R package hierfstat v0.5.11 (Goudet 2005). Wilcoxon signed-rank tests were conducted to test for overall differences in *H*_*o*_ and *H*_*e*_ across sampled localities.

Pairwise population differentiation (*F*_*ST*_) was calculated using hierfstat v0.5.11 (Goudet 2005). Clustering was assessed with the Bayesian approach STRUCTURE (Pritchard et al. 2000). While STRUCTURE can produce misleading results when applied to datasets exhibiting a continuous pattern of genetic differentiation (Bradburd et al. 2018), its application here is justified due to the absence of genuine isolation-by-distance (IBD) patterns (see sections ‘Results’ and ‘Discussion’). MCMC iterations were completed with a burn-in of 10,000 and a further 100,000 repeats for parameter inference. Between 1 and 20 potential clusters (*K*) were investigated, with ten replicate runs per value of *K*. Multiple runs for each *K* were consolidated and the most likely number of genetic clusters was assessed using the Evanno method (Evanno et al. 2005). As this method only detects the uppermost level of structure when several hierarchical levels exist, the analyses were repeated for each inferred cluster separately to test for substructure within clusters (Coulon et al. 2008; Evanno et al. 2005). Localities were assigned to a cluster if the average assignment-value (*q*) was ≥0.6. Genetic differentiation was visualised using principal coordinate analysis (PCoA) using the R package ade4 v1.7-22 (Dray and Dufour 2007). Missing genotype data were imputed by replacing them with the mean allele value at the corresponding locus. We performed an Analysis of Molecular Variance (AMOVA) with 1000 permutations to assess the genetic differentiation between the main genetic clusters obtained in the first STRUCTURE run, using the pegas R package (Paradis 2010).

#### Estimating genetic connectivity

We assessed patterns of IBD across all localities and for each genetic cluster on the first hierarchical *STRUCTURE* level separately. We calculated the chord distance *D*_*c*_ (Cavalli-Sforza and Edwards 1967) as a measure of genetic distance. This measure does not rely on biological assumptions and is generally the most appropriate genetic distance metric for detecting IBD when it exists (Séré et al. 2017). Geographical distances between sampling sites were calculated using Euclidean distances. To test whether river distance better explains genetic differentiation than straight-line geographic distance, we also assessed IBD using pairwise water way distances, which were calculated using the riverdist v0.17.1 package in R (Tyers 2024). To assess the correlation between the geographic and genetic distance matrices, a Mantel test with 999 permutations was conducted using the R package dartR v2.9.7 (Gruber et al. 2018).

We mapped resistance to ongoing dispersal using a spatially explicit, individual-based approach implemented in the R package ResDisMapper (Tang et al. 2020) This method is comparable to other approaches such as DResD and EEMS, but is less computationally intensive and more accurate, particularly in the presence of dispersal barriers (Tang et al. 2020). ResDisMapper is suitable for geographically continuous populations at small spatiotemporal scales, without the need for prior knowledge on environmental features. First, a non-linear IBD model was constructed based on the Prevosti’s genetic distance and Euclidean geographic distance, as this combination of distance matrices resulted in the best-fit with the data at hand. ResDisMapper calculates IBD residuals for each pair of individuals and maps resistance values based on their deviations from the overall IBD trend. To assess the sensitivity of our results across different spatial scales, resistance values and their corresponding significance were calculated between pairs of individuals within a maximum distance of 2, 5, and 10 km (Fig. S5), by randomly resampling IBD residuals in each grid cell 1000 times without replacement. Statistically significant resistance values were visualised as raster layers with two colours depicting significantly lower and higher than expected gene flow, potentially representing dispersal corridors or barriers, respectively (Tang et al. 2020).

To estimate the directionality and relative magnitude of gene flow between sampled localities, the divMigrate algorithm (Sundqvist et al. 2016) was used. This method was preferred over other algorithms as it is less computationally intensive and not affected by prior assumptions (Sundqvist et al. 2016). The divMigrate algorithm uses asymmetric distributions of allele frequencies to quantify gene flow directionality between pairs of localities and provides relative migration rates. These rates range from 0, indicating no migration, to 1, representing substantial migration (Sundqvist et al. 2016). The *Nm*_*Alcala*_ statistic was applied as a measure of genetic differentiation to estimate the relative migration coefficients between localities (Alcala et al. 2014). By incorporating complementary information from both *G*_*st*_ and *D*_*c*_, *Nm*_*Alcala*_ is an appropriate measure of migration, especially when underlying assumptions to quantify effective migration rates are not met (Alcala et al. 2014; Sundqvist et al. 2016). The obtained pairwise relative migration rates (Table S2) were used to calculate the mean relative immigration (*I*) and emigration (*E*) rate per locality, as well as their ratio (*R*_*I/E*_). Differences in *R*_*I/E*_ values between the genetic clusters on the first hierarchical *STRUCTURE* level were tested using a Mann–Whitney *U* test. To assess the presence of source-sink dynamics between sampled localities, linear models were used to examine the relationship between the Euclidean distance from source location (as predicted by geoprofiling analyses), and *R*_*I/E*_ values. This analysis was conducted for all source locations combined, as well as separately for the two initial source locations (i.e., source locations 1 and 3). In the case of the latter, population 31 was identified as an influential outlier and was consequently removed from the dataset.

We further quantified genetic relatedness (i.e., kinship) between all pairs of individuals using the estimator proposed by Wang (2002) with the R package related v1.0 (Pew et al. 2012). Pairs of individuals collected from the same locality with a kinship coefficient of ≥0.5 were considered full-sibs, which can potentially bias genetic parameters (Goldberg and Waits 2010). Pairs of individuals from different localities with a kinship coefficient ≥0.35 were visualised as they are likely to indicate recent dispersal events (Iacchei et al. 2013).

To further explore contemporary dispersal dynamics, we assessed the presence of genetic clines—gradual spatial changes in genetic diversity that can arise from processes such as serial founder effects. Specifically, we examined whether the spatial distribution of *H*_*o*_ was influenced by predicted source locations identified through geoprofiling analyses. For this purpose, we employed the WINGEN v2.1.2 R package, which integrates a sliding window approach with kriging interpolation to estimate *H*_*o*_ across unsampled areas, while controlling for sampling intensity through rarefaction (Bishop et al. 2023). The study area was divided into 5292 raster cells, and a continuous map of *H*_*o*_ was generated using a 10 × 10 cell sliding window. Interpolated Ho values were then extracted at each sampled locality and paired with corresponding hit scores from the geoprofiling output (where lower hit scores indicate higher likelihood of a source). We tested for a statistical association between *H*_*o*_ and hit scores using a Pearson correlation.

## Results

### Citizen-science occurrence records

**Fig. 2:**
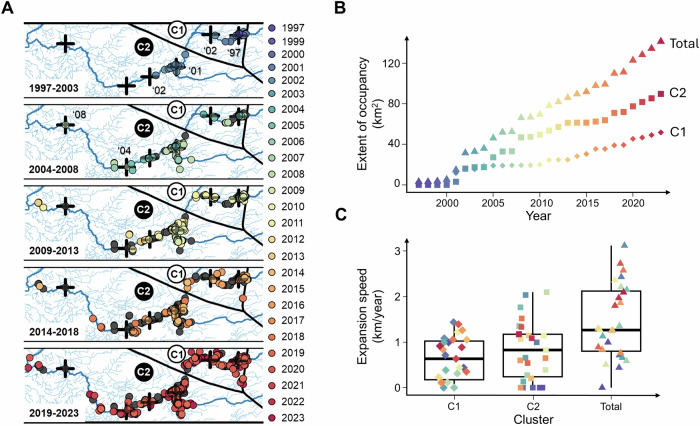
Spatiotemporal invasion dynamics of American bullfrogs in the Grote Nete river valley based on occurrence records. **A** The expansion of the invasion through time, as reflected by the time and location of observation records. Observations are grouped into five-year intervals and colour-coded by year to highlight annual increments. Observations from an earlier interval are indicated in grey. The year-per-year occupancy data suggest that the invasion originated from two main clusters, which are further denoted as the northeastern (NE) and southwestern (SW) cluster. Black crosses mark source locations predicted by the geoprofiling algorithm (Fig. 1), with the year of the first observation near each predicted source location shown. **B** Extent of occupancy (EOO; in km^2^) and **C** range expansion speed (in km per year) are given per year, for both clusters separately and for the total Grote Nete river valley. Triangles represent the total river valley, squares the SW cluster, and diamonds the NE cluster.

Geoprofiling provided the strongest support for six source locations (i.e., regions within the invaded range from which invasive spread most likely began; Fig. 1C). Two source locations were situated upstream and four downstream of the westward extending branch of a canal (Fig. S2). Based on the assumptions made to construct the prior distribution, the DPM model estimated that the mean posterior value of the standard deviation in dispersal distance (sigma) was 1.05 km, with a range between 0.81 and 1.40 km, during the period from 1997 and 2009 (Fig. 1B). For this period, the model thus predicts that, on average, the majority of natural bullfrog dispersal (68%, 95%, and 99%) occurred within approximately 1, 2, and 3 km of their source location, corresponding to an estimated dispersal rate of 0.083, 0.167, and 0.250 km/year, respectively. Temporal increments in occurrence records showed that the invasion began with two clusters, one northeast (NE) and one southwest (SW) from the westward extending branch of a canal (Figs. 2A and S2). From these two initial clusters, new occurrence records primarily emerged in a downstream direction, but the two clusters never completely merged. The DPM model predicted that the NE cluster has originated from two source locations (circa 1997 and 2002), while the SW cluster has stemmed from four source locations (2001, 2002, 2004, and 2008; Fig. 2A). The overall area occupied was greater in the SW cluster (89.68 km^2^ in 2023) compared to the NE cluster (51.69 km^2^ in 2023; Fig. 2B). Consequently, bullfrogs exhibited a faster spread in the SW (818 ± 635 metres per year; average ± standard deviation) than the NE cluster (631 ± 469 metres per year).

### Genome-wide SNP data

Across the 372 individuals analysed, 8592 SNPs were recovered that met the filtering criteria. Kinship analysis identified 94 putative full-sib pairs, from which one random individual per pair was removed, resulting in a final dataset of 278 individuals. The observed heterozygosity (*H*_*o*_) in the sampled localities ranged from 0.174 to 0.259, with an average value of 0.216 (Table 1). The average observed heterozygosity at each sampled locality did not differ significantly from the expected heterozygosity (*V* = 179.000, *p* = 0.417). The inbreeding coefficient (*F*_*IS*_) varied between −0.300 and 0.119, with an average of −0.0186 ± 0.0994. Pairwise *F*_*ST*_ varied between 0.0126 and 0.474 (0.233 on average), revealing two distinct groups: localities 1–18 and 19–31 (Fig. S6).

#### Population genetic structure

**Fig. 3:**
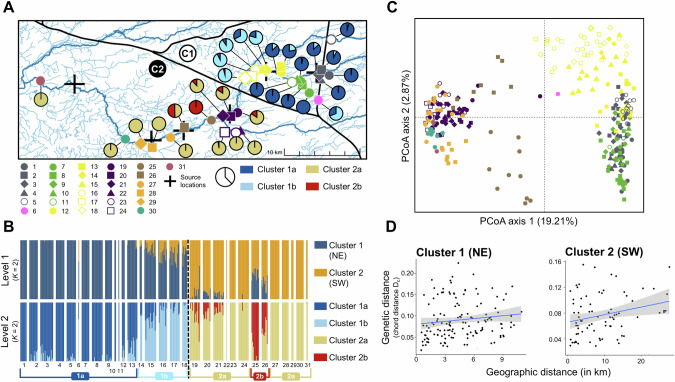
Population genetic structure of localities invaded by American bullfrogs in the Grote Nete river valley using 8592 SNPs (Table 1). **A** Map of the sampled localities with unique colour-coded symbols representing different localities, each associated with a unique locality number (1–31). The average cluster assignments (*q*-value) from the hierarchical STRUCTURE analysis are depicted as pie charts. Predicted source locations from the geoprofiling analyses are marked by black crosses (Fig. 1). **B** Hierarchical STRUCTURE bar plots representing two clusters (*K* = 2) at the first hierarchical level (upper panel), and two clusters (*K* = 2) for each of the resulting clusters at the second hierarchical level (lower panel). **C** Principal coordinate analysis (PCoA) biplot of SNP genotypes per locality, using identical colours and symbols as in (**A**). **D** Patterns of Isolation-By-Distance (IBD) for the NE and SW clusters separately.

At the first hierarchical level, the most likely number of genetic clusters was two (*K* = 2; Fig. S7), and these clusters were located on opposite sides of the westward extending branch of the canal. Cluster 1 (NE) comprised localities 1–18 and was located in the northeastern part of the invaded area. Cluster 2 (SW) comprised localities 19–31 and was located downstream from cluster 1, in the southwestern part of the invaded area (Figs. 3A, B and S2, S7). At the second hierarchical level, the NE cluster was subdivided into two subclusters (*K* = 2; Fig. S7). The first subcluster (1a) included localities 1–13 and was situated more upstream, while the second subcluster (1b) comprised localities 14–18. Similarly, the SW cluster comprised two subclusters (*K* = 2) at the second hierarchical level. Subcluster 2b included localities 25 and 26, and was positioned within the geographically broader subcluster 2a, consisting of localities 19–24 and 27–31.

The PCoA revealed patterns consistent with those observed in the STRUCTURE analysis (Fig. 3C). A distinct separation between localities 1–18 (NE cluster) and 19–31 (SW cluster) was observed along the first Principal Coordinate (PCoA) axis. Subclusters 1a and 1b could be discriminated along the second PCoA axis, roughly reflecting their geographical position in the Grote Nete river valley. Subcluster 2a and 2b were also distinguishable, with subcluster 2a exhibiting more extreme values for PCoA axis 1 than subcluster 2b. The AMOVA results indicated significant genetic differentiation between the NE and SW clusters (*Φ*_*ST*_ = 0.308, *p* < 0.001), with 30.8% of the total genetic variation attributable to differences between these main clusters. IBD was not observed among NE localities (*r* = 0.098, *p* = 0.19) or SW localities (*r* = 0.260, *p* = 0.119; Fig. 3D). However, evidence of IBD was found across all localities in the Grote Nete river valley (*r* = 0.574, *p* = 0.001). The IBD analyses based on water way distance yielded similar patterns to those using Euclidean distance (Fig. S8).

#### Genetic connectivity

**Fig. 4:**
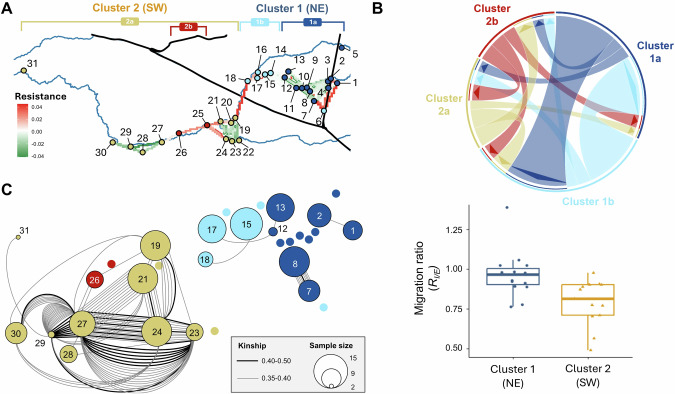
Gene flow patterns between sampled localities invaded by American bullfrogs in the Grote Nete river valley, clustered and coloured according to the hierarchical STRUCTURE analysis. **A** Ongoing fine-scale resistance to gene flow, as determined by ResDisMapper, was assessed using pairs of sampled individuals within a 5 km radius. Output values ranged from −1 (green) to 1 (red), indicating higher (corridor) and lower (barrier) than expected gene flow, respectively. **B** Directionality and relative magnitude of gene flow between clusters obtained through STRUCTURE analysis. Boxplots show the distribution of the migration ratio (*R*_*I/E*_) for localities in the NE and SW clusters separately. **C** Pairwise relatedness between individuals of different sampled localities. Thick black and thin grey lines represent pairs of individuals with a Wang’s kinship estimator between 0.40–0.50 and 0.35–0.40, respectively. The size of sampled localities was scaled according to the total number of sampled individuals. Localities without any highly related individuals are given as small circles without a locality number. Localities are jittered for visualisation purposes.

Mapping of IBD residuals indicated discontinuous gene flow between localities (Fig. 4A). Barriers to gene flow (i.e., significant positive IBD residuals) were observed between localities on opposite sides of the canals (i.e., between locality 1 and localities 2–7 for the northward extending canal branch, and subclusters 1b and 2a for the westward extending branch), between localities near and those located further away from the river (between locality 6 and localities of cluster 1a), and downstream of the confluence with the Laak river (between localities 19–24 and 25–26; Fig. S2). Aside from these barriers, localities along the Grote Nete river were generally associated with significantly negative IBD residuals, suggesting gene flow is facilitated (Fig. 4A). These patterns remained consistent regardless of whether resistance was calculated using sampled pairs within grids of 2 km, 5 km, or 10 km (Fig. S5).

Substantial variation in the directionality and magnitude of gene flow was observed across localities. Estimated rates of relative directional gene flow ranged from 0.0331 to 1 with an average of 0.167 (Table S2). The highest rates of relative directional gene flow were associated with localities 2 and 3 in the NE cluster, and localities 19, 20, 21, and 27 in the SE cluster. With an immigration to emigration (*R*_*I/E*_) ratio <1, these localities potentially produced more migrants than they received, similar to most other sampled localities (Table 1). Conversely, localities 1, 7, 13, and 15 potentially received more migrants than they produced. The relative immigration to emigration ratio was significantly higher in the NE (0.966 ± 0.144) than the SW (0.791 ± 0.143) cluster (*W* = 29.5, *p* = 0.002). We found no significant relationship between the distance to the nearest source, as predicted by geoprofiling, and the *R*_*I/E*_ ratio. This was the case both when considering all source locations (*t* = −0.855, *p* = 0.400) and when calculating the distance to only the two initial source locations (*t* = −1.750, *p* = 0.092).

Overall, gene flow was higher in the SW cluster compared to the NE cluster, mainly occurring among subclusters, and in both upstream as downstream directions (Fig. 4B). Within the SW cluster, and particularly within cluster 2a, localities shared a large number of closely related individuals (Fig. 4C). Even locality 31, which was situated substantially further downstream than the other localities, contained an individual that was highly related with an individual from locality 29.

**Fig. 5:**
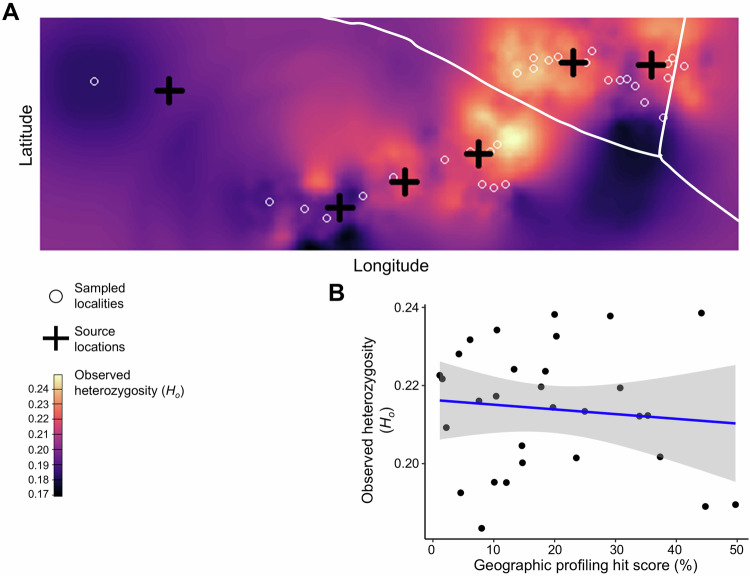
Assessment of geographic clines in genetic diversity of American bullfrogs in the Grote Nete river valley in relation to source locations predicted by geoprofiling. **A** Map of observed heterozygosity (*H*_*o*_), corrected for varying sampling size through rarefaction. Dark and light colours represent low and high *H*_*o*_ values, respectively. Black crosses mark source locations predicted by the geoprofiling algorithm (Fig. 1). **B** Relationship between the hit score (i.e., the proportion of the area covering the source locations) obtained from geoprofiling and the interpolated *H*_*o*_ value. Lower hit scores indicate a higher probability of the presence of a source location.

Although some localised hotspots and coldspots of *H*_*o*_ were detected, no clear spatial clines were observed across the study area (Fig. 5A). There was no significant correlation between *H*_*o*_ and the hit scores derived from geoprofiling analyses (*t* = −0.546, *p* = 0.589; Fig. 5B).

## Discussion

Characteristic of bullfrog introductions to Europe is their origin from personal initiatives and unregulated pet trade, leading to a poorly documented invasion history (Ficetola et al. 2007). The bullfrog invasion in the Grote Nete river valley is commonly believed to involve a single, continuous population established by one introduction followed by downstream dispersal (Descamps and De Vocht 2023). By combining geoprofiling based on citizen science occurrence records with detailed population genetic analyses, our study challenges this scenario, revealing a complex pattern of multiple primary and secondary introductions combined with natural short-distance and long-distance dispersal (LDD) events.

### Multiple source locations

Geoprofiling predicted that bullfrogs likely started to spread from six source locations (Fig. 1C). Additionally, population genetics identified two main genetic clusters on each side of a canal (Fig. 3A, B), suggesting that at least two of these source locations were founded by primary introductions (Sard et al. 2023). This was further supported by spatiotemporal analyses of occurrence records, highlighting two clusters (i.e., NE and SW) marking the onset of the invasion (Fig. 2A). The first observation in the NE cluster was recorded in 1997 at a fish farm (near source location 1), while a complex of cottages (near source location 3), many of which featuring a pond, marked the first observation in the SW cluster in 2001. These findings support anecdotal evidence of two introduction pathways: as hitchhikers via fish imports and through pet trade (Jooris 2005).

Hierarchical genetic clustering analyses further divided each main genetic cluster into two subclusters (Fig. 3A, B), suggesting that additional processes, beyond primary introductions, contribute to the observed genetic structure (Coulon et al. 2008). In the NE cluster, two source locations were predicted by geoprofiling (numbers 1 and 2), roughly aligning with the location of the two genetic subclusters. In addition, a trend of gradually increasing proportions of cluster 1b genotypes in a downstream direction was observed (Fig. 3B). Such spatially non-random admixed genetic profiles are likely to result from natural dispersal (Kamath et al. 2016; Garroway et al. 2011). Source location 1 may be the result of a primary introduction event (Fig. 2A). Downstream source location 2 has been established by individuals genetically comparable to those from source location 1 (Fig. 3A, B), through either a primary introduction of similar genetic provenance, a secondary introduction, or LDD events from upstream localities (cf. geoprofiling), followed by natural dispersal from source location 1 (cf. population genetics and occurrences).

While the SW cluster comprised two genetic subclusters, geoprofiling analyses indicated four source locations (numbers 3–6). The occurrence of LDD events is one of the factors potentially explaining this discrepancy. During periods of extreme precipitation, the Grote Nete river can overflow its banks near source location 3 (Fig. S9), inundating extensive areas and facilitating passive downstream spread (Mims et al. 2015). Some source locations (e.g., numbers 5 and 6) may therefore be founded by individuals passively spreading from upstream source locations (number 3), as suggested by kinship (i.e., many closely related individuals; Fig. 4C) and gene flow (i.e., high rate of emigration; Fig. 4D) analyses. The occurrence of LDD events is further supported by the lack of strong serial founder effects (Garroway et al. 2011; Berthouly-Salazar et al. 2013), as indicated by the absence of a correlation between observed heterozygosity (*H*_*o*_) and geoprofiling hit scores (Fig. 5). Similar dynamics drive the bullfrog invasion in the Yellowstone river valley (Sepulveda et al. 2015), but also other biological invasions (Mothes and Searcy 2024). Due to their genetic similarity, source locations founded by LDD events are unlikely to be uncovered using population genetic structuring, as they are grouped into a single genetic cluster (Ficetola et al. 2008; Ryan et al. 2019).

Multiple primary or secondary introductions of genetically similar individuals (source locations 2 and 4) could also have contributed to this mismatch between geoprofiling analyses and population genetic structure. While source locations founded by such introductions are difficult to differentiate using genetic data alone (Estoup et al. 2010; Ryan et al. 2019), they can be identified using geoprofiling, provided that the locations are sufficiently spaced apart relative to the species’ dispersal ability (Verity et al. 2014). Nonetheless, multiple primary and secondary introductions are common for species traded as pets (Sinclair et al. 2021). The high rate of bidirectional gene flow between localities across the NE and SW clusters (Fig. 4) support the occurrence of multiple secondary introductions, whether these are signals reflect past or recent events.

Although Flanders is one of the most densely populated regions in Europe—suggesting relatively even observation effort (Swinnen et al. 2018)—we acknowledge that the discrepancy between analyses based on both data types could also have originated due to spatial biases in occurrence records. However, the DPM method used for geoprofiling performs equally well with opportunistic occurrence records, which are often affected by spatial biases, as it does with systematic survey data (Faulkner et al. 2017). Additionally, part of the records used for geoprofiling were collected by nature organisations with the specific goal of mapping the initial distribution of bullfrogs (Jooris 2005). We therefore consider it unlikely that this discrepancy is an artefact of spatial biases in occurrence data. Collectively, these explanations underline the complementarity of analysing both genetic and occurrence data (Lu et al. 2023; Sherpa and Després 2021).

### Rapid, linear, and discontinuous range expansion

The invaded area primarily expanded downstream along the river at a consistent rate, averaging 1.45 km per year (Fig. 2B, C). The range expansion stage is therefore linear rather than exponential, as is commonly observed (Haubrock et al. 2022). Exponential range expansion rates are often driven by the depletion of suitable habitats, which forces individuals to disperse further, thereby accelerating the rate of spread (Berthouly-Salazar et al. 2013; Fraser et al. 2015). Nonetheless, linear range expansion rates are typical for species exhibiting patchy distributions comprising multiple source locations located close enough to allow for short-term coalescence (Shigesada and Kawasaki 2016). Indeed, bullfrog spread resembled discontinuous leading-edge range expansions (Fig. 2A) where populations from different source locations within each cluster merged over time (Wilson et al. 2009). This spread pattern is supported by the lack of a significant relationship between distance from the source location and the immigration-to-emigration ratio (i.e., indicative of source-sink dynamics between core and edge populations; Micheletti and Storfer 2020), or between the geoprofiling hit score and the observed heterozygosity (i.e., indicative of absent serial founder effects; Garroway et al. 2011).

Despite the coalescence of the majority of source locations over time, NE and SW clusters remained spatially and genetically isolated. This discontinuity may be attributed to the surrounding landscape, where a canal, with the river culverted beneath it, runs between both clusters. In addition, a highway and an industrial area run parallel to the westward-extending canal (Fig. S10). Such landscape features are known to obstruct amphibian gene flow (Van Buskirk 2012), and may collectively act as dispersal barriers (Peterson et al. 2013). Unlike both the Yellowstone river valley and prevailing assumptions, the bullfrog populations invading the Grote Nete river valley may therefore not be a single continuous, interconnected population (Kamath et al. 2016). Regardless, the observed range expansion shows no indication of approaching a saturation stage (Fig. 2B), either overall or within each cluster, suggesting that the invasion is not yet approaching carrying capacity and that bullfrogs are likely to continue spreading.

The observed expansion rate is in line with comparable biological invasions. For instance, African clawed frogs (*Xenopus laevis*) spread with a consistent speed of 1.2 km per year in France (Pagano et al. 2024) and signal crayfish (*Pacifastacus leniusculus*) most commonly spread up to 2 km per year in Western European countries (Hudina et al. 2009). Our results suggest that the rapid spread of bullfrogs in the Grote Nete river valley can be explained by multiple source locations established through primary or secondary introductions, which initiated range expansions at several localities simultaneously, combined with LDD events facilitating rapid downstream spread. However, range expansion speed can vary independently of dispersal capacity, influenced by factors such as prevailing environmental conditions, the density of breeding habitats, and the intensity of management efforts (Fraser et al. 2015; Urban et al. 2008). Additionally, the pace of expansion can change over the course of range expansions due to evolutionary processes (Phillips et al. 2006). The comparatively warmer climate, more natural environment, and lower management intensity in the Yellowstone river valley may therefore have contributed to the faster range expansion of bullfrogs (15 km per year) compared to that along the strongly anthropogenically modified Grote Nete river, where eradication programs have been in place for over a decade (Sepulveda et al. 2015; Descamps and De Vocht 2023). Alternatively, spatially differential selective pressures can lead to an increased investment in reproduction (*r*-selection) near invasion fronts, whereas selection pressures favouring increased investment in competitive ability (*K*-selection) can be expected in the core distribution range (Burton et al. 2010). However, it is unlikely that evolutionary forces are already affecting spread dynamics of bullfrogs in Belgium due to the recent nature of the invasion and the relatively slow generation time (Courant et al. 2019). Moreover, the high degree of gene flow observed at the invasion front might swamp local adaptation (Beer et al. 2024), preventing these eco-evolutionary processes from occurring.

### Asymmetric and bidirectional gene flow

Our results indicated an overall isolation-by-distance (IBD) pattern, likely resulting from the westward extending canal branch impeding bullfrog dispersal (Fig. 1C). No significant IBD pattern was detected when tested within clusters on either side of this barrier separately (Fig. 3C). Given the high dispersal capacity of bullfrogs (Smith and Green 2005) and the limited observed genetic differentiation across geographic distances (Fig. S6), the absence of IBD within clusters further supports the occurrence of natural dispersal, stochastic LDD events, or multiple primary or secondary introductions (Hutchison and Templeton 1999; Berthouly-Salazar et al. 2013; Mothes and Searcy 2024). This suggests that secondary contact between multiple introductions, rather than an equilibrium between gene flow and genetic drift, established the observed population genetic structure (Bingham et al. 2021). Indeed, similar to the high levels of migration in the native range of bullfrogs (Austin et al. 2004), bullfrogs in the Grote Nete river valley were generally found to experience low dispersal resistance, reflecting its high invasibility (Everts et al. 2023; Vimercati et al. 2024). Multiple primary introductions and high degree of post-introduction dispersal leading to genetic admixture potentially explains not only the rapid spread rates, but also the lack of observable reduced heterozygosity or inbreeding due to isolation of local demes (Kolbe et al. 2008).

While the invaded range primarily expanded in a downstream direction along the Grote Nete river (Fig. 2A), gene flow occurred in both directions (Fig. 4B). Although asymmetric migration was also observed in their native range (Austin et al. 2004), bidirectional migration is not common in nature, particularly in systems driven by physical transport processes like river currents (Pringle et al. 2011). Together with the similar patterns of IBD obtained using both water way or Euclidean distances (Fig. S8), this suggests that dispersal in the Grote Nete river valley might occur independent of river current (Mazerolle 2005), and that overland movements or secondary introductions might have contributed to bullfrog spread (Peterson et al. 2013). Gene flow mainly occurred between localities on the same side of the westward extending canal branch (Fig. 4), supporting its role as a barrier. Moreover, SW localities were more likely to receive than to send out individuals, and contained a larger number of closely related individuals relative to NE localities (Fig. 4B, C). These spatially heterogenous gene flow patterns may explain the substantially faster range expansion in the SW compared to NE cluster (Fig. 2B, C). We expect that bullfrogs in SW localities may continue to expand along the river valley, as there are no apparent dispersal barriers and plenty of suitable habitats further downstream, while frequently inundated wetlands will likely continue to promote passive downstream spread (Fig. S9; Mims et al. 2015; Sepulveda et al. 2015). Conversely, further spread in the NE cluster may be restricted by canals crossing the river at both the upstream and downstream invasion fronts. We therefore expect bullfrogs in the NE cluster to enter the saturation stage soon (Andow et al. 1990), unless they alter their dispersal behaviour by moving beyond the river valley and no longer following the path of least resistance (i.e., the more favourable or accessible habitats) offered by the river valley (Trumbo et al. 2016; Vimercati et al. 2024). The disproportionately large number of closely related individuals between localities 7 and 8 suggests this is already occurring (Fig. 4C).

### Conclusion

By integrating occurrence record analyses with population genetics, our study provides novel insights into the invasion history of bullfrogs in the Grote Nete river valley in Belgium and the factors governing their spread dynamics. Our results showed that the bullfrog invasion in the Grote Nete river valley in Belgium originated from multiple source locations followed by bidirectional dispersal, challenging the parsimonious scenario of a single primary introduction followed by downstream dispersal (Descamps and De Vocht 2023). Furthermore, multiple source locations combined with inundation-driven LDD events likely contributed to the observed rapid range expansion. Our findings further reveal a dispersal barrier shaping invasion dynamics. With the growing availability and quality of large-scale occurrence records (Jarić et al. 2020) and population genetic data (Lancaster et al. 2023), their combined use will become invaluable for advancing our understanding of expanding biological invasions.

## Supplementary information

Supporting Information 1

Supplementary figure and tables

## Data Availability

Data and code used for analyses are available in Zenodo (10.5281/zenodo.15240192). Similarly, the raw sequences generated in the present study have been deposited in the European Nucleotide Archive (ENA) at EMBL-EBI under accession number PRJEB88153.
