# Supplementary material for: Multiple source locations and long-distance dispersal explain the rapid spread of a recent amphibian invasion
Source: Heredity (Edinb). 2025 May 16;134(6):362–73. doi: 10.1038/s41437-025-00766-w (PMC12137657; doi:10.1038/s41437-025-00766-w)
Supplement: Supplementary file 1 — Supporting Information 1 [file 41437_2025_766_MOESM1_ESM.docx]

Supporting information 1 for:

**Multiple source locations and long-distance dispersal explain the rapid spread of a recent amphibian invasion**

**Journal name**: Heredity

# Restriction enzyme selection

The selection of restriction enzymes and the final size selection step were based on simulated *in silico* genome digestions using the SimRAD R package v0.96 (Lepais & Weir, 2014), and a bullfrog reference genome (Hammond et al., 2017). A total of 11 enzyme combinations were tested *in silico* (Table S1). Six combinations produced the desired intermediate number of size-selected fragments (250-350 bp; Christiansen et al., 2021), and were subsequently tested *in vitro*. For this purpose, ten bullfrog tissue samples from ten ponds spread across the distribution range were selected. Six replicates for each sample were prepared, resulting in 60 libraries following the protocol outlined below. The combination *PstI* – *EcoRI* yielded the best result in terms of the fragment size distribution (determined using Qiagen’s QIAXcel) and the number of fragments generated, and was used for the remaining samples.

# Library preparation

The GBS libraries were prepared following the protocol established by Elshire et al. (2011) and Poland et al. (2012), which is briefly explained below. Seven pools, each containing up to 96 individual libraries, were prepared. These pools included 372 individuals, 14 blank samples, and 42 replicates from 12 individuals. A total of 100 ng of DNA per sample was used whenever possible. Individual DNA samples were digested with restriction enzymes *PstI* and *EcoRI* at 37°C for 2 hours. A unique barcoded adaptor (*PstI*) and a common adaptor (*EcoRI*) were then ligated to the digested DNA at 22°C for 2 hours, followed by incubation at 65°C for 20 minutes and subsequent cooling to 4°C. All samples were purified using 1.6X Ampure XP beads (Beckman Coulter) and eluted in final volume of 30 µl. Then, 3 µl of the purified DNA fragments was amplified through 18 cycles of PCR, with each cycle consisting of 30, 20, and 30 seconds at 96, 65, and 68°C, respectively. During this amplification step, unique and pool-specific Illumina i5 and i7 adapters were added to the fragments. The obtained fragments were purified using 1.6X Ampure XP beads and eluted in a volume of 25 µl. The final concentration of each library was quantified using a Promega Quantus™ fluorimeter. All samples from the seven pools were combined and included 10 ng from each individual library. This final pool, including all 428 samples, was then purified (1.6X Ampure XP beads) and sequenced 150 bp paired-end on an Illumina NovaSeq S4 instrument (Admera Health NV, New Jersey).

# Bioinformatic data processing

The quality of the raw reads was initially validated using FastQC v0.11 (Andrews, 2010). Preprocessing of the raw reads was carried out with GBprocesS v4.0.0 (Schaumont, 2020). Raw reads were first demultiplexed with Cutadapt v4.3 (Martin, 2011, ensuring an exact barcode match. Next, Cutadapt was used to remove cutsite remnants and adapter sequences from the demultiplexed reads. Paired reads with an overlap of at least 10 bp and a minimum length of 40 bp were merged using PEAR v0.9.11 (Zhang et al., 2014). Quality control of the merged reads involved several filters: reads containing at least one N nucleotide were discarded (MaxNFilter), reads were removed if any of two consecutive bases had an average Phred quality below 20 (SlidingWindowQualityFilter), and reads with an overall average Phred quality below 25 were omitted (AverageQualityFilter). Finally, reads with intact internal restriction enzyme recognition sites were removed.

The remaining reads were aligned to the American bullfrog reference genome available on the NCBI GenBank platform (Accession No. GCA_002284835.2; Hammond et al., 2017, using the BWA-MEM algorithm within *BWA* v0.7.17 (Li, 2013. Reads with multiple matches and a MAPQ score below 20 were discarded. Picard v2.25.1 (Picard Toolkit, 2019) was used to index each BAM file generated by BWA and incorporate read groups. The proportion of successfully mapped reads ranged from 50.1% to 98.4%, with an average of 88.5%. Of these successfully mapped reads, 33.6% to 49.3% (averaging 46.5%) had a MAPQ score greater than 20 and were retained.

The Genome Analysis Toolkit (GATK) v4.2.0.0 (McKenna et al., 2010) was used to detect SNPs within the mapped reads. Initially, SNPs were called for each individual using the HaplotypeCaller from GATK, resulting in one GVCF file per individual. Subsequently, all GVCF files were consolidated into a single file using the CombineGVCF tool, after which the GenotypeGVCF tool was applied to genotype all the samples collectively. Then, the SelectVariants tool was utilized to only retain biallelic loci, and the obtained SNPs were filtered using the VCFtools software v0.1.16 (Danecek et al., 2011). A minimum sequencing depth of 10 was required for SNPs to be retained (--min-meanDP 10), and singletons were filtered out (--mac 3, --maf 0.03). Then, SNPs and genotypes with a quality of 20 (--minQ 20) and 30 (--minGQ 30) or less, respectively, were omitted, as well as genotypes supported by less than 10 reads (--minDP 10.0). Only polymorphic SNPs were retained. Finally, SNPs and individuals with a missingness of ≥20% and a mean read depth of >100 were removed, as well as SNPs with an observed heterozygosity (*H_o_*) of >0.5. No filtering was conducted based on departure from Hardy-Weinberg equilibrium (HWE), as this could result in a reduced ability to detect population structure (Pearman et al., 2022. Independence between SNPs was assessed based on pairwise analysis of linkage disequilibrium (LD; ­-indep-pairwise 2 1 0.5) in which one of two linked SNPs are randomly excluded if an LD correlation factor (*r*^2^) >0.5 is obtained.

# References

Andrews, S. (2010). *FastQC a Quality Control Tool for High Throughput Sequence Data*. https://www.bioinformatics.babraham.ac.uk/projects/fastqc/

Christiansen, H., Heindler, F. M., Hellemans, B., Jossart, Q., Pasotti, F., Robert, H., Verheye, M., Danis, B., Kochzius, M., Leliaert, F., Moreau, C., Patel, T., Van de Putte, A. P., Vanreusel, A., Volckaert, F. A. M., & Schön, I. (2021). Facilitating population genomics of non-model organisms through optimized experimental design for reduced representation sequencing. *BMC Genomics*, *22*(625). https://doi.org/10.1186/s12864-021-07917-3

Danecek, P., Auton, A., Abecasis, G., Albers, C. A., Banks, E., DePristo, M. A., Handsaker, R. E., Lunter, G., Marth, G. T., Sherry, S. T., McVean, G., Durbin, R., & 1000 Genomes Project Analysis Group. (2011). The variant call format and VCFtools. *Bioinformatics*, *27*(15), 2156–2158. https://doi.org/10.1093/bioinformatics/btr330

Elshire, R. J., Glaubitz, J. C., Sun, Q., Poland, J. A., Kawamoto, K., Buckler, E. S., & Mitchell, S. E. (2011). A robust, simple genotyping-by-sequencing (GBS) approach for high diversity species. *PLoS ONE*, *6*(5), e19379. https://doi.org/10.1371/journal.pone.0019379

Hammond, S. A., Warren, R. L., Vandervalk, B. P., Kucuk, E., Khan, H., Gibb, E. A., Pandoh, P., Kirk, H., Zhao, Y., Jones, M., Mungall, A. J., Coope, R., Pleasance, S., Moore, R. A., Holt, R. A., Round, J. M., Ohora, S., Walle, B. V, Veldhoen, N., … Birol, I. (2017). The North American bullfrog draft genome provides insight into hormonal regulation of long noncoding RNA. *Nature Communications*, *8*(1433). https://doi.org/10.1038/s41467-017-01316-7

Lepais, O., & Weir, J. T. (2014). SimRAD: an R package for simulation-based prediction of the number of loci expected in RADseq and similar genotyping by sequencing approaches. *Molecular Ecology Resources*, *14*, 1314–1321. https://doi.org/10.1111/1755-0998.12273

Li, H. (2013). Aligning sequence reads, clone sequences and assembly contigs with BWA-MEM. *ArXiv*, *1303.3997*.

Martin, M. (2011). Cutadapt removes adapter sequences from high-throughput sequencing reads. *EMBnet.Journal*, *17*(1), 10–12. https://journal.embnet.org/index.php/embnetjournal/article/view/200

McKenna, A., Hanna, M., Banks, E., Sivachenko, A., Cibulskis, K., Kernytsky, A., Garimella, K., Altshuler, D., Gabriel, S., Daly, M., & DePristo, M. A. (2010). The genome analysis toolkit: A MapReduce framework for analyzing next-generation DNA sequencing data. *Genome Research*, *20*(9), 1297–1303. <https://doi.org/10.1101/gr.107524.110>

Picard Toolkit. 2018. Broad Institute, GitHub Repository. https://github.com/broadinstitute/picard

Pearman, W. S., Urban, L., & Alexander, A. (2022). Commonly used Hardy–Weinberg equilibrium filtering schemes impact population structure inferences using RADseq data. *Molecular Ecology Resources*, *22*(7), 2599–2613. https://doi.org/10.1111/1755-0998.13646

Poland, J. A., Brown, P. J., Sorrells, M. E., & Jannink, J.-L. (2012). Development of high-density genetic maps for barley and wheat using a novel two-enzyme genotyping-by-sequencing approach. *PLoS ONE*, *7*(2), e32253. https://doi.org/10.1371/journal.pone.0032253

Schaumont, D. (2020). *GBprocesS: genotyping-by-sequencing data processing toolkit*.

Zhang, J., Kobert, K., Flouri, T., & Stamatakis, A. (2014). PEAR: A fast and accurate Illumina Paired-End reAd mergeR. *Bioinformatics*, *30*(5), 614–620. https://doi.org/10.1093/bioinformatics/btt593
