# Supplementary material for: Multiple source locations and long-distance dispersal explain the rapid spread of a recent amphibian invasion
Source: Heredity (Edinb). 2025 May 16;134(6):362–73. doi: 10.1038/s41437-025-00766-w (PMC12137657; doi:10.1038/s41437-025-00766-w)
Supplement: Supplementary file 2 — Supplementary figure and tables [file 41437_2025_766_MOESM2_ESM.docx]

Supporting information for:

**Multiple source locations and long-distance dispersal explain the rapid spread of a recent amphibian invasion**

**Journal name**: Heredity

**Figure S1**. Total number of expert-verified bullfrog occurrence records in Belgium stored in GBIF per year (downloaded on November 3, 2023; [doi.org/10.15468/dl.zj25kf](https://doi.org/10.15468/dl.zj25kf)). The bars represent the annual number of bullfrog occurrence records (lefthand y-axis), while the dots indicate the cumulative number of observations (righthand y-axis). The first officially recorded occurrence of bullfrog in Flanders (northern region of Belgium) dates back to 1996. Initially, the number of occurrence records remained low, primarily originating from nature organizations and conservationists. In 2006, the citizen-science paltform “waarnemingen.be”, a regional adaptation of “observations.org”, was launched, enabling citizens to submit species observations online. This led to an increase in yearly bullfrog observations. In 2009, the species Decree legally prohibited deliberate releases of bullfrogs. By setting the upper limit of occurrence records used for geoprofiling to 2009 (indicated by black bars), we (i) ensured that widely spaced occurrence records originated from additional introductions rather than natural dispersal from undocumented sites, and (ii) expanded the spatial and temporal scope of bullfrog observations by including three years of expert-verified citizen-science records. All available, expert-verified occurrence records located in the Grote Nete river valley were used to analyze the occupied area and range expansion speed.

**Figure S2**. Map of the 31 sampled bullfrog locations in the Grote Nete valley in Belgium retained for molecular analyses. The grey polygons outlined by black dotted lines indicate the distribution of bullfrogs in Belgium, as reported by Everts et al. (2023).

**Figure S3.** The sampling strategy for the genetic study. Since the genetic structure of a complete population per pond must be estimated from a subset of individuals, we designed a sampling strategy that approximated the true genetic diversity in a pond as closely as possible. (A) At least two fyke nets were installed in each pond, positioned as far apart as possible. Single fyke nets were placed perpendicular to the pond’s bank to maximize the likelihood of capturing juveniles and adults in addition to tadpoles, as depicted by the number 1 in the figure. Double fyke nets were installed parallel to and approximately two meters from the bank (number 2 on the figure), maximizing the likelihood of capturing tadpoles. The number of fyke nets was adjusted according to the size of the pond, with a maximum of five fyke nets per pond. All fyke nets were emptied approximately 24 hours after placement. Sampled life stages are shown in the lower half of the figure. (B) Hypothetical example of the catch per fyke net when 2 fyke nets were used, where the numbers and silhouettes correspond to the fyke nets and life stages in (A), respectively. When more than 20 individual bullfrogs were captured in total (upper panel), individuals were selected based on two criteria: (i) maximizing diversity in life stages (i.e. not only sampling first-year tadpoles, but also second-year tadpoles, juveniles and adults), and (ii) pursuing an equal contribution from the catches of multiple fyke nets to represent the population sample of the pond. When fewer than 20 total individuals were captured in total (lower panel), all captured individuals were sampled. Tadpoles were sampled by tail clipping, while juveniles and adults were sampled by toe clipping. Icons are from cleanpng.com. Pictures of life stages courtesy of Teun Everts.

**Figure S4.** Estimating the degree of relatedness between all pairs of individuals. (A) Pairwise correlation between the most commonly used metrics of relatedness, calculated using the coancestry function of the *relatedness* R-package. Given that the Wang estimator has been shown to outperform other metrics (Wang 2007), and that there were strong correlations among estimators in our dataset, we chose to focus on the Wang estimator for further analyses. The relatedness metrics used are as follows: Wang = relatedness estimate described by Wang (2002); lynchli = estimate described by Li et al. (1993); lynchrd = estimate described by Lynch and Ritland (1999); ritland = estimate described by Ritland (1996); quellergt = estimate described by Queller and Goodnight (1989); and dyadml = dyadic likelihood estimator, described by Milligan (2003). (B) Density plot showing the frequency distribution of the Wang estimator values for each pair of individuals in the study. Pairs of individuals with a Wang estimator value exceeding 0.5 (indicated by the red dotted line) are considered full-sibs. One random individual from each of these pairs was subsequently removed from the dataset.

**Figure S5**. Output of *ResDisMapper* for three distance thresholds used to model resistance to gene flow: (A) 2 km, (B) 5 km, and (C) 10 km. Sampling points are shown as black dots. Red areas indicate high resistance to gene flow, while green areas indicate low resistance. Contour lines delineate regions of statistically significant high (red) or low (green) resistance, respectively. Cells with no statistical certainty are masked in white. Although the maximum reported annual dispersal capacity of invasive bullfrogs is estimated to be around 2 km per year (Smith & Green 2005), we chose to model resistance to gene flow between locations separated by up to 5 km, due to the discontinuous sampling across the river valley. The Grote Nete river and its tributaries are shown in blue, while the northward and westward extending branches of the canal crossing the river are depicted in black.

**Figure S6.** Pairwise *F_ST_* values for all sampled bullfrog locations. Pairs of locations with higher values of *F_ST_* are represented with darker colors, indicating greater genetic differentiation between locations. No *F_ST_* values are available for locations 6, 11 or 22, as only one individual per location was included in the final dataset.

**Figure S7.** Assessment of the most optimal number of genetic clusters (K), using the Evanno method. Based on the delta K plots (upper plots) and mean lnP(K) plots (lower plots), the optimal number of clusters was determined to be two. When a second STRUCTURE analysis was run on both obtained clusters separately, the most optimal number of clusters was also two in both cases. Although the lnP(K) plots suggested tgat higher optimal K-values might be optimal, increasing K led to difficulties in assigning individuals and locations to specific clusters, as *q*-values did not reach the threshold of ≥ 0.6

**Figure S8**. Patterns of Isolation-By-Distance (IBD), using the chord distance as a measure of genetic distance. Geographic distance is represented by Euclidean distance (upper panels) and waterway distance (lower panels). The plots are shown for locations in the NE cluster (left), locations in the SW cluster (center), and all locations combined (right).

**Figure S9**. Map of the flood risk zones in the study area (indicated in blue), derived from a combination of modeled flood areas and recently inundated areas, adjusted using the Digital Elevation Model of Flanders. During periods of extreme precipitation, the Grote Nete river can overflow its banks, inundating extensive areas and potentially facilitating passive downstream spread. The likelihood of this occurring is higher in the Southwestern cluster (cluster 2) located downstream of the westward extending branch of the canal (highlighted in light blue) compared to the Northeastern cluster (cluster 1). The black arrow indicates a frequently inundated area that is likely to have contributed to the downstream spread of invasive bullfrogs. The inset on the right-hand side shows an aerial photograph of the area indicated by the black arrow during the winter flooding of 2023-2024 (courtesy of Dieder Plu). This map was provided by Flanders Environmental Agency, and can be downloaded from https://www.vlaanderen.be/datavindplaats/catalogus/risicozones-overstromingen-2017

**Figure S10**. Map depicting the percentage build-up area (shown in black) within the study area. The Grote Nete River is highlighted in dark blue, the canal in light blue, and the highway in red. A photograph of the culvert channeling the Grote Nete river beneath the canal is shown in the right-hand side (courtesy of Teun Everts), and an aerial image of the industrial terrain is shown in the left-hand side (courtesy of Google Earth). This map was provided by Agency Digital Flanders, and can be downloaded from https://download.vlaanderen.be/product/7681-bodemafdekkingskaart_(bak)_5m_resolutie_opname_2018.

**Table S1.** Results of the *in silico* digestion by restriction enzymes using a bullfrog draft genome reference sequence of all combinations between either *EcoRI* or *PstI* with *MseI*, *MspI*, *ApekI*, *HindIII* and each other. Fragment generation with each pair of restriction enzymes was conducted with a random 10% subsample of the genome, and was repeated ten times. Average and standard deviation of the total number of generated fragments, and fragments with lengths between 250 and 350 bp are given. In the case that an enzyme combination generated too few fragments of the desired length, total percentage coverage of the genome decreases, together with the total number of SNPs identified. Conversely, when too many fragments of desired length are generated, read depth per fragment decreases, requiring more total sequencing depth per sample to capture all loci. Only samples with an intermediate number of fragments with lengths between 250 and 350 bp were therefore used for further *in vitro* testing.

**Table S2.** Results of the *DivMigrate* analysis, using the *Nm_Alcala_* statistic as a measure of population genetic differentiation. Upper half (shaded in grey) represents the migration rates into the sampled locations (immigration) whereas the lower half shows the emigration rates for each sampled location. For instance, the rate of immigration from all locations to location 1 can be found in the first row, while the rate of emigration from location 1 to all other sampled locations can be found in the first column. Locations 32 was omitted as it was geographically isolated compared to other locations, as well as locations 6, 11, and 22 due to limited sampling size.

**Figure S1**


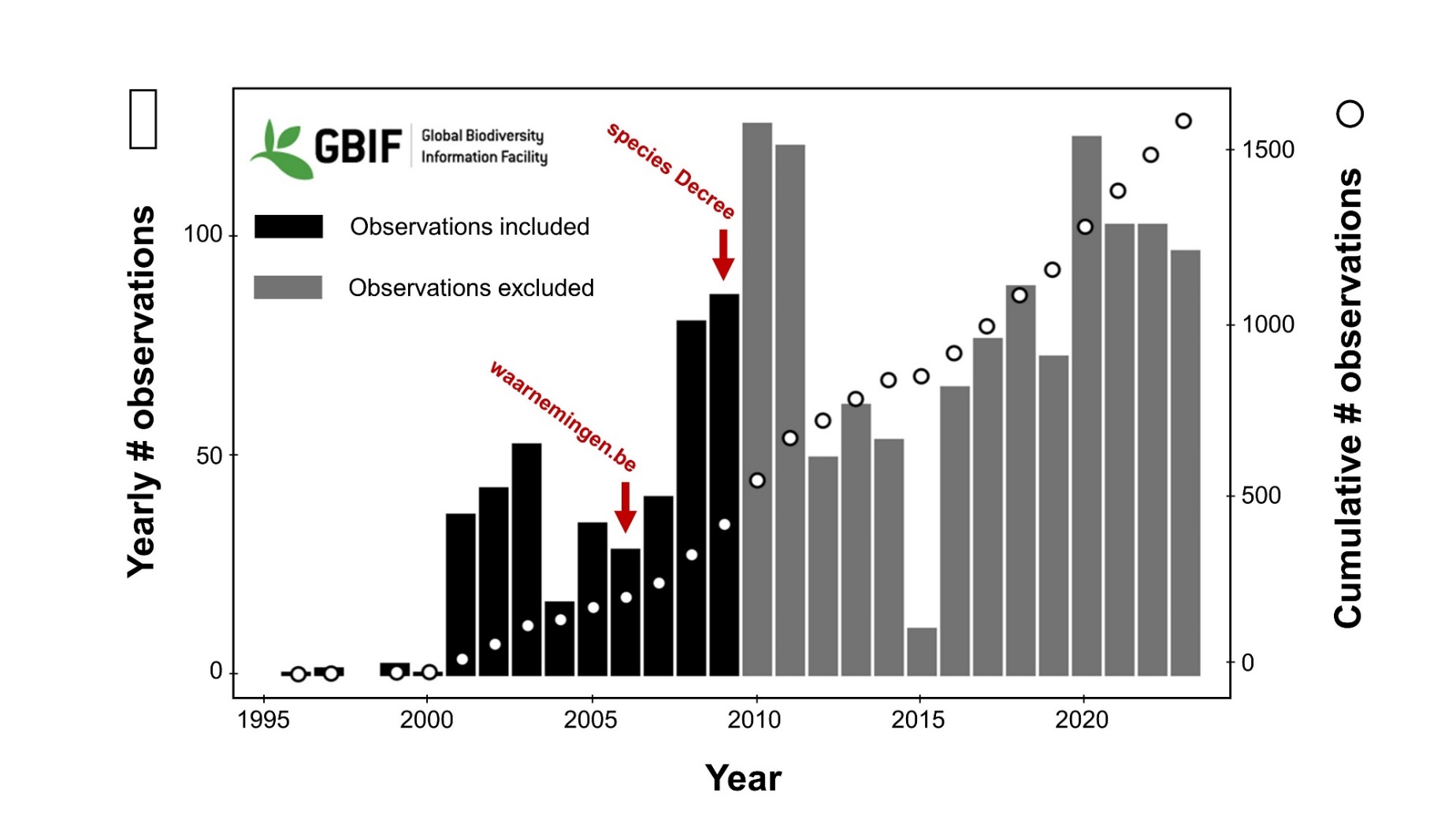


**
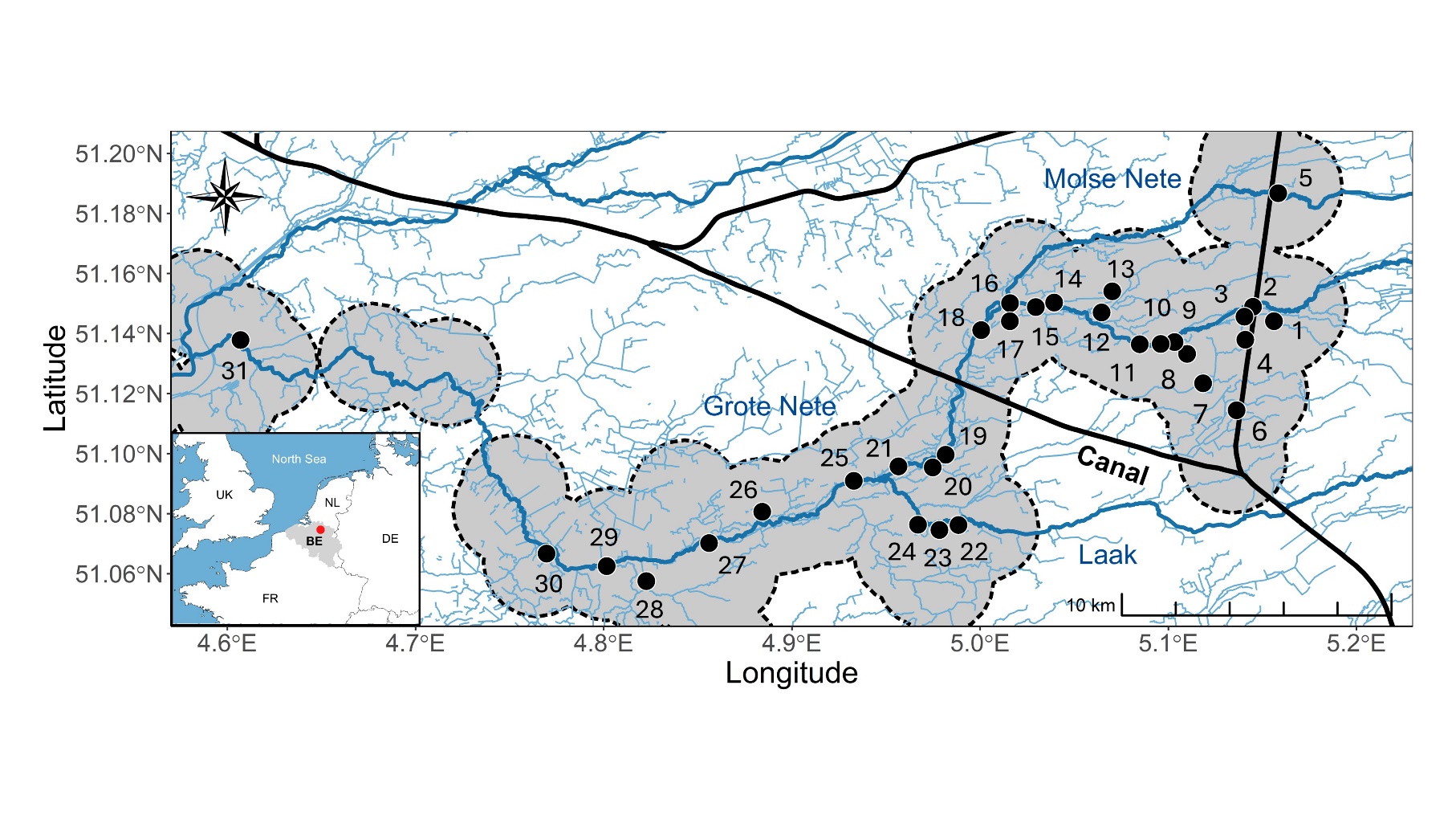
Figure S2**

**Figure S3**


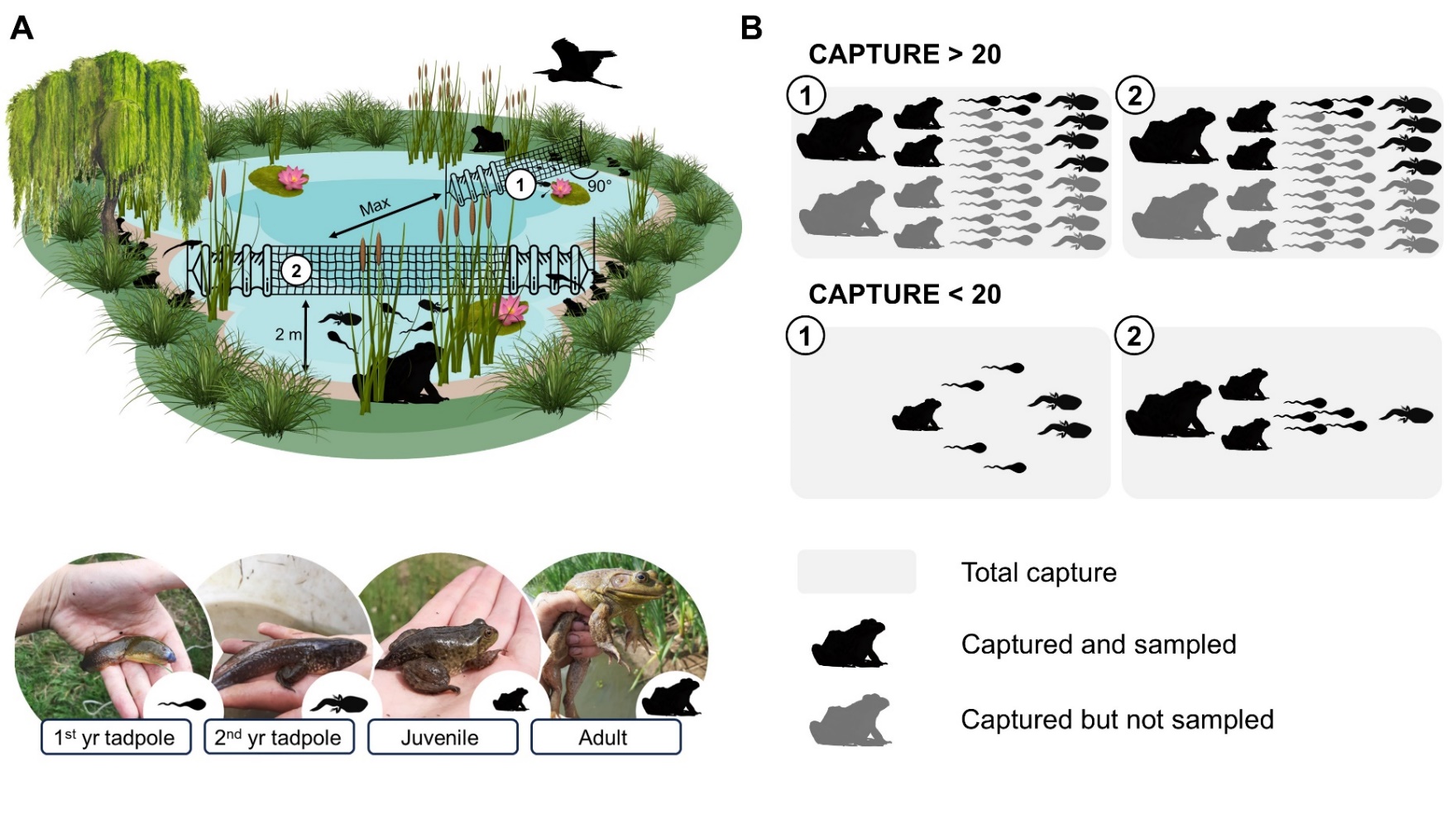


**Figure S4**


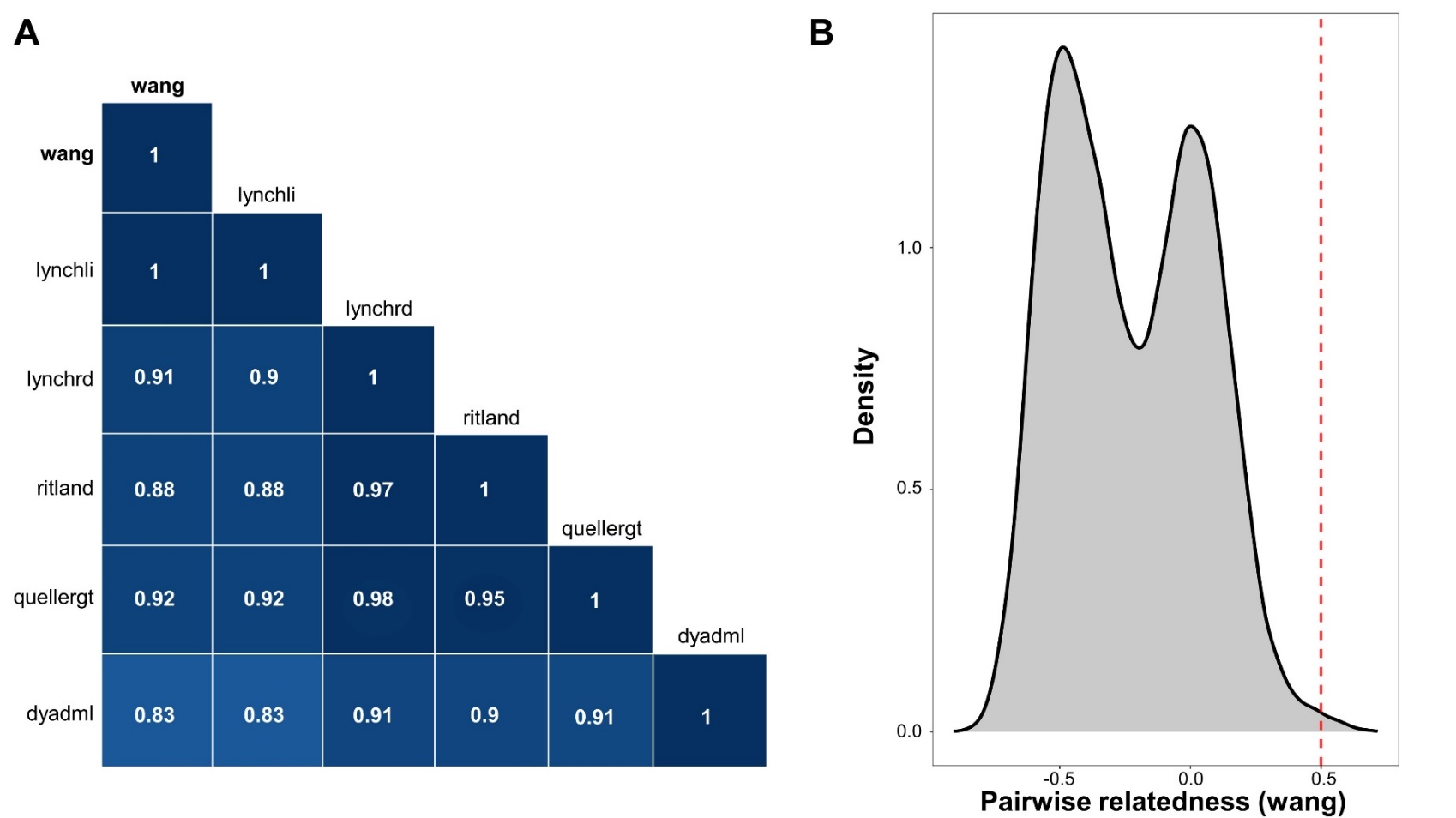


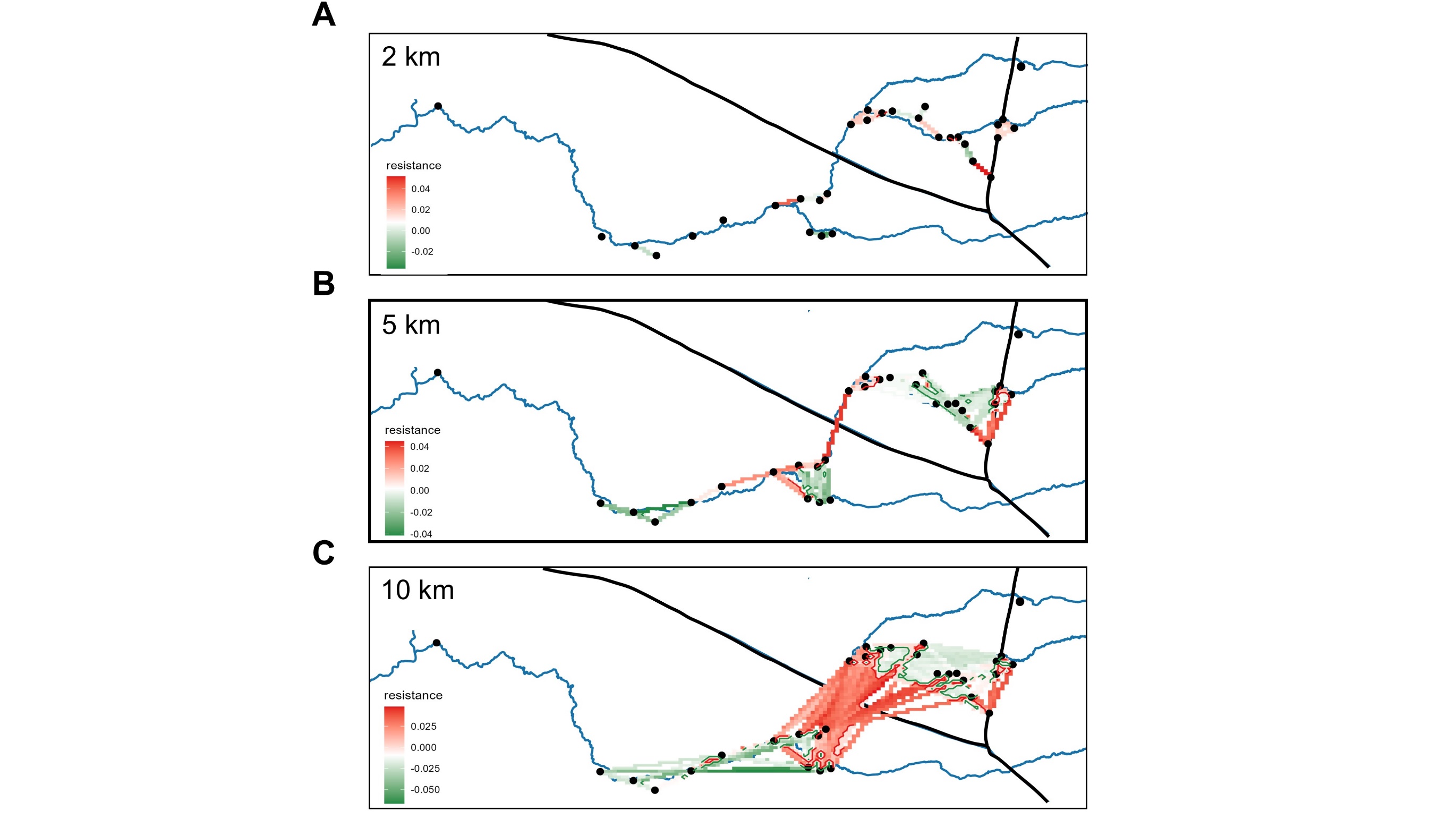
**Figure S5**


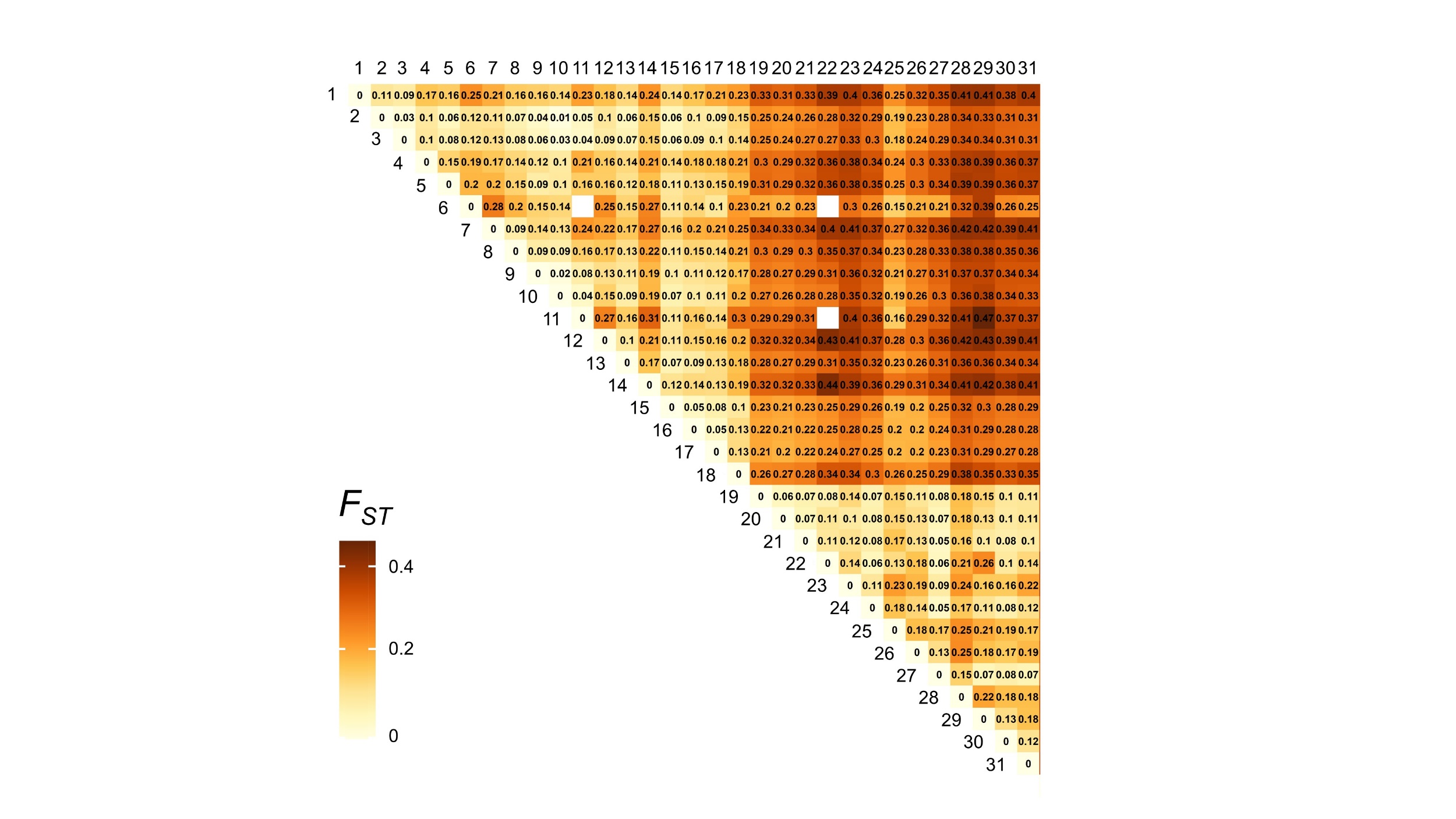
**Figure S6**

**Figure S7**

**
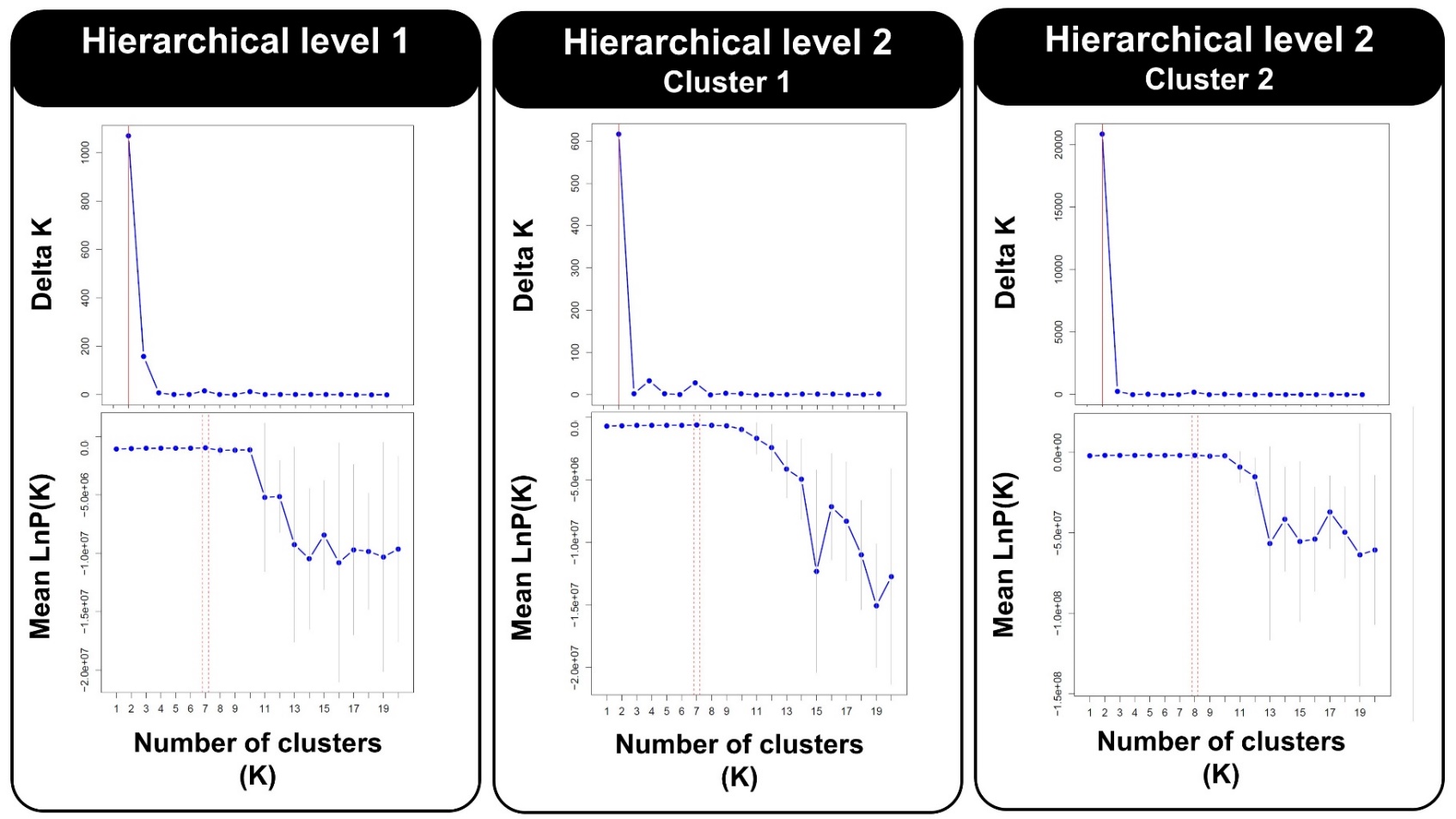
**

**Figure S8**

**
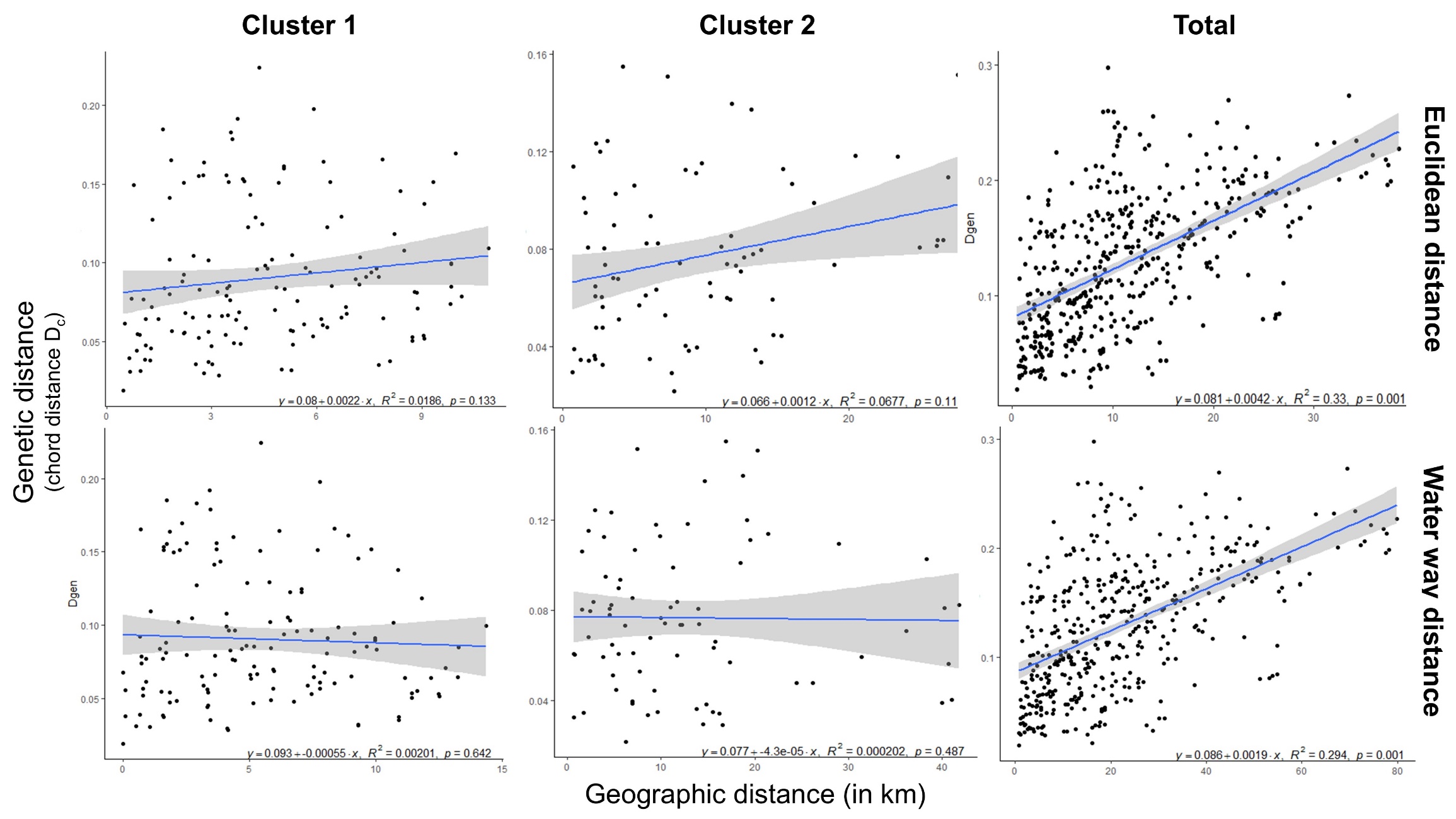
**


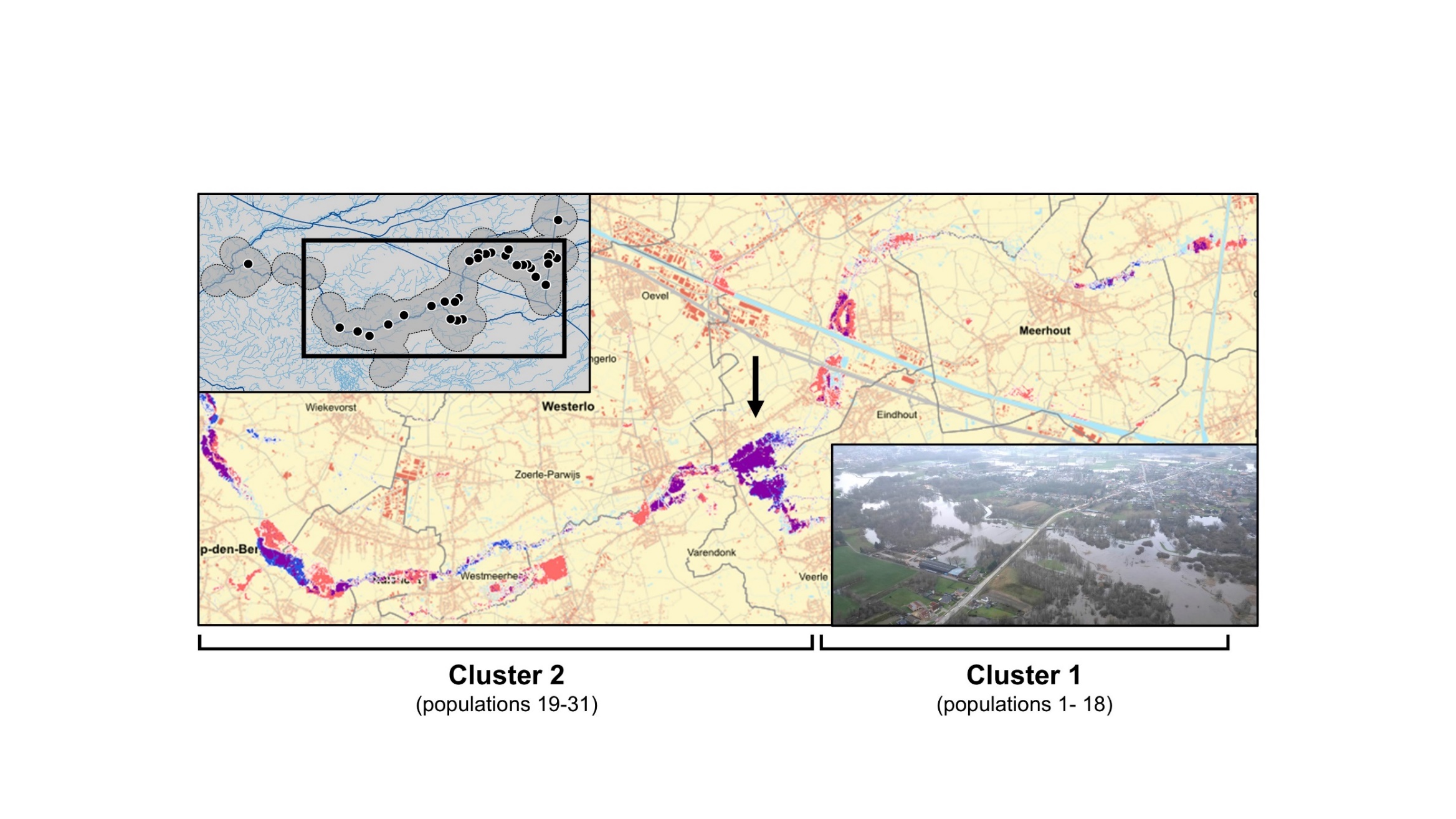
**Figure S9**

**
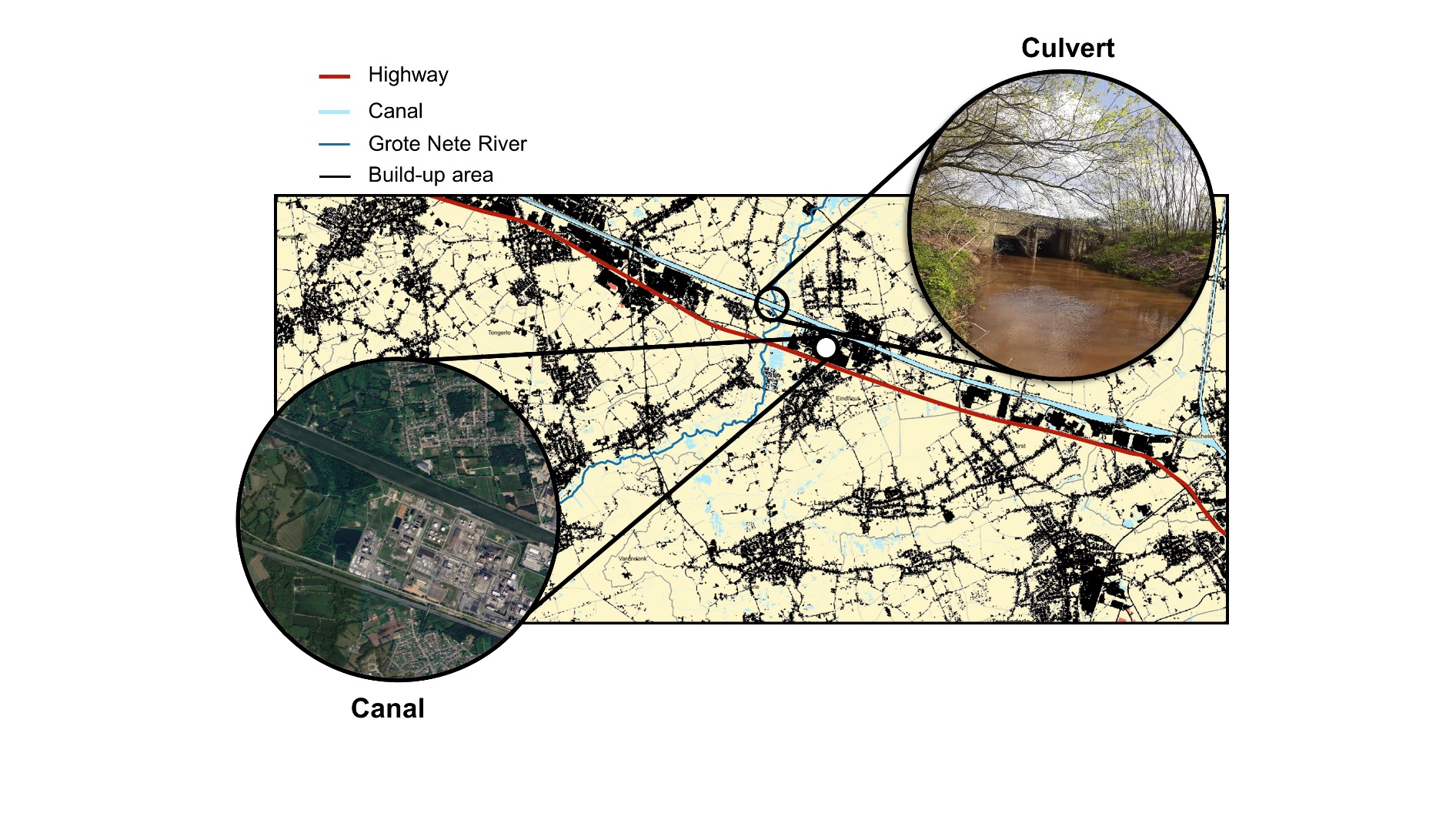
Figure S10**

**Table S1**

| Enzyme 1 | Enzyme 2 | Total fragment number | Number of size-selected fragments (250-350 bp) | *In vitro* testing? |
| --- | --- | --- | --- | --- |
| *PstI* | *PstI* | 1 114 438 ± 17 092 | 54 032 ± 1 258 | Yes |
| *PstI* | *MspI* | 1 309,308 ± 17 782 | 112 836 ± 1 683 | No, too many |
| *PstI* | *MseI* | 1 788 475 ± 17 479 | 178 310 ± 2 536 | No, too many |
| *PstI* | *HindIII* | 1 443 670 ± 29 305 | 90 371 ± 1 974 | Yes |
| *PstI* | *ApeKI* | 137 250 ± 5 749 | 12,189 ± 394 | No, too few |
| *PstI* | *EcoRI* | 526 325 ± 8 569 | 28 674 ± 486 | Yes |
| *EcoRI* | *EcoRI* | 388 188 ± 3 451 | 8 928 ± 284 | No, too few |
| *EcoRI* | *MspI* | 612 868 ± 9 056 | 45 625 ± 988 | Yes |
| *EcoRI* | *MseI* | 724 518 ± 11 372 | 67 154 ± 1 015 | Yes |
| *EcoRI* | *HindIII* | 434 804 ± 6 981 | 19 587 ± 604 | Yes |
| *EcoRI* | *ApeKI* | 187,321 ± 3 946 | 13 509 ± 376 | No, too few |

**Table S2**

|  | **1** | **2** | **3** | **4** | **5** | **7** | **8** | **9** | **10** | **12** | **13** | **14** | **15** | **16** | **17** | **18** | **19** | **20** | **21** | **23** | **24** | **25** | **26** | **27** | **28** | **29** | **30** | **31** |
| --- | --- | --- | --- | --- | --- | --- | --- | --- | --- | --- | --- | --- | --- | --- | --- | --- | --- | --- | --- | --- | --- | --- | --- | --- | --- | --- | --- | --- |
| **1** | - | 0.439 | 0.582 | 0.203 | 0.219 | 0.139 | 0.242 | 0.217 | 0.089 | 0.128 | 0.272 | 0.078 | 0.269 | 0.193 | 0.160 | 0.111 | 0.100 | 0.076 | 0.077 | 0.050 | 0.062 | 0.113 | 0.072 | 0.059 | 0.048 | 0.040 | 0.056 | 0.038 |
| **2** | 0.212 | - | 1.000 | 0.274 | 0.423 | 0.193 | 0.405 | 0.527 | 0.147 | 0.165 | 0.485 | 0.091 | 0.524 | 0.326 | 0.342 | 0.146 | 0.142 | 0.113 | 0.105 | 0.063 | 0.081 | 0.148 | 0.099 | 0.077 | 0.056 | 0.048 | 0.070 | 0.045 |
| **3** | 0.245 | 0.953 | - | 0.289 | 0.359 | 0.187 | 0.393 | 0.486 | 0.143 | 0.184 | 0.470 | 0.096 | 0.545 | 0.337 | 0.320 | 0.164 | 0.145 | 0.114 | 0.103 | 0.063 | 0.081 | 0.158 | 0.097 | 0.076 | 0.058 | 0.048 | 0.070 | 0.045 |
| **4** | 0.149 | 0.464 | 0.462 | - | 0.209 | 0.148 | 0.266 | 0.297 | 0.117 | 0.130 | 0.302 | 0.079 | 0.250 | 0.199 | 0.197 | 0.103 | 0.107 | 0.083 | 0.078 | 0.050 | 0.065 | 0.107 | 0.070 | 0.061 | 0.048 | 0.040 | 0.056 | 0.039 |
| **5** | 0.181 | 0.721 | 0.715 | 0.242 | - | 0.137 | 0.241 | 0.377 | 0.113 | 0.150 | 0.331 | 0.089 | 0.396 | 0.266 | 0.256 | 0.124 | 0.111 | 0.086 | 0.076 | 0.051 | 0.063 | 0.107 | 0.069 | 0.061 | 0.046 | 0.041 | 0.056 | 0.038 |
| **7** | 0.130 | 0.462 | 0.453 | 0.243 | 0.183 | - | 0.506 | 0.329 | 0.104 | 0.109 | 0.269 | 0.068 | 0.281 | 0.189 | 0.165 | 0.096 | 0.096 | 0.077 | 0.076 | 0.047 | 0.060 | 0.099 | 0.071 | 0.054 | 0.043 | 0.039 | 0.051 | 0.036 |
| **8** | 0.158 | 0.578 | 0.582 | 0.221 | 0.224 | 0.312 | - | 0.437 | 0.115 | 0.120 | 0.320 | 0.076 | 0.346 | 0.242 | 0.238 | 0.113 | 0.114 | 0.089 | 0.088 | 0.054 | 0.067 | 0.122 | 0.074 | 0.065 | 0.048 | 0.042 | 0.059 | 0.040 |
| **9** | 0.167 | 0.718 | 0.673 | 0.253 | 0.347 | 0.182 | 0.325 | - | 0.160 | 0.158 | 0.331 | 0.082 | 0.424 | 0.324 | 0.281 | 0.133 | 0.121 | 0.089 | 0.082 | 0.054 | 0.067 | 0.133 | 0.081 | 0.062 | 0.048 | 0.041 | 0.059 | 0.039 |
| **10** | 0.130 | 0.465 | 0.430 | 0.179 | 0.169 | 0.128 | 0.227 | 0.313 | - | 0.107 | 0.233 | 0.079 | 0.259 | 0.193 | 0.182 | 0.088 | 0.095 | 0.080 | 0.073 | 0.048 | 0.057 | 0.100 | 0.068 | 0.055 | 0.045 | 0.038 | 0.051 | 0.038 |
| **12** | 0.139 | 0.410 | 0.503 | 0.187 | 0.185 | 0.103 | 0.214 | 0.241 | 0.087 | - | 0.356 | 0.074 | 0.352 | 0.227 | 0.200 | 0.114 | 0.106 | 0.074 | 0.071 | 0.044 | 0.055 | 0.084 | 0.067 | 0.052 | 0.042 | 0.035 | 0.048 | 0.035 |
| **13** | 0.178 | 0.636 | 0.642 | 0.209 | 0.276 | 0.140 | 0.282 | 0.305 | 0.105 | 0.201 | - | 0.088 | 0.527 | 0.360 | 0.261 | 0.125 | 0.126 | 0.096 | 0.089 | 0.053 | 0.071 | 0.118 | 0.086 | 0.066 | 0.049 | 0.040 | 0.060 | 0.038 |
| **14** | 0.109 | 0.265 | 0.268 | 0.155 | 0.168 | 0.103 | 0.149 | 0.158 | 0.082 | 0.122 | 0.206 | - | 0.313 | 0.253 | 0.261 | 0.124 | 0.097 | 0.071 | 0.070 | 0.047 | 0.059 | 0.078 | 0.068 | 0.056 | 0.044 | 0.038 | 0.050 | 0.036 |
| **15** | 0.174 | 0.522 | 0.613 | 0.218 | 0.284 | 0.154 | 0.289 | 0.314 | 0.113 | 0.163 | 0.471 | 0.119 | - | 0.611 | 0.435 | 0.233 | 0.155 | 0.125 | 0.119 | 0.067 | 0.088 | 0.145 | 0.111 | 0.085 | 0.060 | 0.048 | 0.073 | 0.043 |
| **16** | 0.137 | 0.333 | 0.398 | 0.164 | 0.216 | 0.121 | 0.213 | 0.240 | 0.105 | 0.138 | 0.354 | 0.111 | 0.653 | - | 0.598 | 0.196 | 0.158 | 0.122 | 0.115 | 0.069 | 0.092 | 0.136 | 0.108 | 0.086 | 0.057 | 0.049 | 0.074 | 0.042 |
| **17** | 0.108 | 0.374 | 0.372 | 0.162 | 0.204 | 0.122 | 0.235 | 0.249 | 0.100 | 0.132 | 0.287 | 0.118 | 0.501 | 0.599 | - | 0.193 | 0.167 | 0.131 | 0.126 | 0.074 | 0.097 | 0.140 | 0.110 | 0.091 | 0.061 | 0.054 | 0.081 | 0.046 |
| **18** | 0.099 | 0.276 | 0.295 | 0.150 | 0.166 | 0.103 | 0.151 | 0.182 | 0.075 | 0.116 | 0.218 | 0.096 | 0.413 | 0.314 | 0.289 | - | 0.125 | 0.087 | 0.092 | 0.052 | 0.068 | 0.097 | 0.085 | 0.064 | 0.045 | 0.038 | 0.058 | 0.033 |
| **19** | 0.052 | 0.089 | 0.093 | 0.068 | 0.067 | 0.053 | 0.072 | 0.077 | 0.045 | 0.049 | 0.088 | 0.048 | 0.142 | 0.159 | 0.140 | 0.110 | - | 0.626 | 0.462 | 0.211 | 0.520 | 0.226 | 0.239 | 0.332 | 0.119 | 0.113 | 0.308 | 0.092 |
| **20** | 0.060 | 0.095 | 0.100 | 0.073 | 0.072 | 0.059 | 0.075 | 0.083 | 0.050 | 0.054 | 0.091 | 0.053 | 0.156 | 0.168 | 0.160 | 0.114 | 0.623 | - | 0.511 | 0.234 | 0.430 | 0.229 | 0.216 | 0.390 | 0.133 | 0.116 | 0.292 | 0.090 |
| **21** | 0.055 | 0.089 | 0.093 | 0.066 | 0.066 | 0.057 | 0.072 | 0.079 | 0.046 | 0.050 | 0.085 | 0.047 | 0.150 | 0.172 | 0.147 | 0.110 | 0.556 | 0.494 | - | 0.218 | 0.491 | 0.209 | 0.230 | 0.554 | 0.156 | 0.145 | 0.434 | 0.098 |
| **23** | 0.045 | 0.065 | 0.069 | 0.052 | 0.051 | 0.043 | 0.054 | 0.059 | 0.036 | 0.040 | 0.062 | 0.041 | 0.106 | 0.123 | 0.116 | 0.087 | 0.339 | 0.472 | 0.395 | - | 0.490 | 0.133 | 0.150 | 0.440 | 0.117 | 0.105 | 0.230 | 0.062 |
| **24** | 0.050 | 0.076 | 0.080 | 0.061 | 0.059 | 0.051 | 0.063 | 0.068 | 0.042 | 0.046 | 0.075 | 0.043 | 0.126 | 0.152 | 0.129 | 0.100 | 0.575 | 0.534 | 0.400 | 0.259 | - | 0.195 | 0.199 | 0.752 | 0.142 | 0.134 | 0.397 | 0.084 |
| **25** | 0.079 | 0.148 | 0.162 | 0.100 | 0.101 | 0.079 | 0.112 | 0.125 | 0.058 | 0.061 | 0.125 | 0.055 | 0.165 | 0.155 | 0.140 | 0.097 | 0.217 | 0.188 | 0.152 | 0.097 | 0.147 | - | 0.125 | 0.137 | 0.082 | 0.071 | 0.121 | 0.062 |
| **26** | 0.066 | 0.116 | 0.116 | 0.079 | 0.081 | 0.068 | 0.089 | 0.095 | 0.055 | 0.064 | 0.108 | 0.057 | 0.177 | 0.188 | 0.169 | 0.125 | 0.356 | 0.257 | 0.270 | 0.129 | 0.231 | 0.167 | - | 0.198 | 0.081 | 0.087 | 0.145 | 0.058 |
| **27** | 0.056 | 0.088 | 0.090 | 0.067 | 0.068 | 0.056 | 0.072 | 0.077 | 0.047 | 0.052 | 0.084 | 0.051 | 0.130 | 0.163 | 0.154 | 0.114 | 0.559 | 0.539 | 0.683 | 0.280 | 0.718 | 0.219 | 0.259 | - | 0.178 | 0.164 | 0.366 | 0.108 |
| **28** | 0.046 | 0.065 | 0.069 | 0.054 | 0.051 | 0.045 | 0.057 | 0.059 | 0.040 | 0.042 | 0.065 | 0.038 | 0.085 | 0.109 | 0.089 | 0.067 | 0.331 | 0.254 | 0.239 | 0.135 | 0.314 | 0.131 | 0.125 | 0.233 | - | 0.076 | 0.217 | 0.081 |
| **29** | 0.044 | 0.061 | 0.063 | 0.050 | 0.050 | 0.043 | 0.052 | 0.055 | 0.035 | 0.040 | 0.059 | 0.040 | 0.089 | 0.102 | 0.098 | 0.082 | 0.292 | 0.281 | 0.368 | 0.177 | 0.362 | 0.148 | 0.166 | 0.516 | 0.132 | - | 0.270 | 0.071 |
| **30** | 0.048 | 0.069 | 0.074 | 0.057 | 0.056 | 0.046 | 0.058 | 0.064 | 0.039 | 0.044 | 0.069 | 0.040 | 0.108 | 0.116 | 0.103 | 0.087 | 0.443 | 0.428 | 0.360 | 0.160 | 0.450 | 0.199 | 0.187 | 0.472 | 0.137 | 0.116 | - | 0.096 |
| **31** | 0.044 | 0.061 | 0.065 | 0.049 | 0.049 | 0.043 | 0.053 | 0.056 | 0.036 | 0.040 | 0.061 | 0.040 | 0.081 | 0.094 | 0.082 | 0.063 | 0.311 | 0.328 | 0.258 | 0.114 | 0.268 | 0.149 | 0.128 | 0.257 | 0.100 | 0.079 | 0.212 | - |

**References**

Everts, T., Van Driessche, C., Neyrinck, S., Jacquemyn, H., & Brys, R. (2023) The American bullfrog exposed: distribution, invasion fronts, and spatial configuration of invasion hubs revealed by eDNA-based monitoring and environmental assessments. *Management of Biological Invasions*, **14**(2), 201–220. <https://doi.org/10.3391/mbi.2023.14.2.02>

Li, C. C., Weeks, D. E. & Chakravarti, A. (1993) Similarity of DNA fingerprints due to chance and relatedness. *Human Heredity* **43**(1): 45-52. https://doi.org/10.1159/000154113.

Lynch, M. & Ritland, K. (1999) Estimation of pairwise relatedness with molecular markers. *Genetics* **152**(4): 1753-1766. <https://doi.org/10.1093/genetics/152.4.1753>.

Milligan, B. G. (2003) Maximum-likelihood estimation of relatedness. *Genetics* **163**(3): 1153-1167. https://doi.org/ 10.1093/genetics/163.3.1153.

Queller, D. C. & Goodnight, K. F. (1989) Estimating relatedness using genetic markers. *Evolution* **43**(2): 258-275. https://doi.org/10.1111/j.1558-5646.1989.tb04226.x.

Ritland, K. (1996) Estimators for pairwise relatedness and individual inbreeding coefficients. *Genetics Research Cambridge Core* **67**: 175-185.

Smith M. A. & Green, D. M. (2005) Dispersal and the metapopulation paradigm in amphibian ecology and conservation: are all amphibian populations metapopulations? *Ecography* **28**: 110–128, https://doi.org/10.1111/j.0906-7590.2005.04042.x

Wang, J. (2002) An estimator of pairwise relatedness using molecular markers. *Genetics* **160**(3): 1203-1215. https://doi.org/10.1093/genetics/160.3.1203.
